# Dietary Interventions and Oral Nutritional Supplementation in Inflammatory Bowel Disease: Current Evidence and Future Directions

**DOI:** 10.3390/nu17111879

**Published:** 2025-05-30

**Authors:** Brigida Barberio, Luisa Bertin, Sonia Facchin, Erica Bonazzi, Sara Cusano, Giulia Romanelli, Francesco Francini Pesenti, Emanuela Cazzaniga, Paola Palestini, Fabiana Zingone, Edoardo Vincenzo Savarino

**Affiliations:** 1 Department of Surgery, Oncology, Gastroenterology, University of Padua, Via Giustiniani 2, 35121 Padua, Italysonia.facchin@unipd.it (S.F.);; 2 Gastroenterology Unit, Azienda Ospedale, Università Padova, 35128 Padua, Italy; 3 Clinical Nutrition Unit, Department of Medicine, University of Padua, 35128 Padua, Italy; 4 School of Medicine and Surgery, University of Milano-Bicocca, 20900 Monza, Italypaola.palestini@unimib.it (P.P.); 5 POLARIS Research Centre, University of Milano-Bicocca, 20900 Monza, Italy

**Keywords:** diet, inflammatory bowel disease, oral nutritional supplementation

## Abstract

Background: Nutritional management has become an integral part of Inflammatory Bowel Disease (IBD) care, with growing evidence supporting specific dietary interventions alongside pharmacologic therapy. However, clinical guidance remains fragmented due to heterogeneous study designs and variable endpoints. Objectives: This review critically examines the current evidence on dietary strategies and oral nutritional supplementation (ONS) in both Crohn’s Disease (CD) and Ulcerative Colitis (UC), highlighting their clinical applications, mechanisms of action, and limitations. Methods: A comprehensive literature search was conducted using PubMed, Scopus, and Web of Science databases, analyzing studies on various dietary approaches and ONS in IBD. Results: Exclusive Enteral Nutrition (EEN) is a first-line therapy in pediatric CD, while partial enteral nutrition (PEN) and the Crohn’s Disease Exclusion Diet (CDED) show promising efficacy and better adherence in both children and adults. Whole-food-based interventions, including the Mediterranean Diet, Specific Carbohydrate Diet, plant-based diets, and emerging strategies such as CD-TREAT and the Tasty & Healthy diet, have demonstrated varying levels of benefit in disease maintenance and symptom control. Targeted exclusion diets—such as low-FODMAP, low-emulsifier, and low-sulfur diets—may relieve functional symptoms and influence inflammatory activity, although evidence remains preliminary. ONS plays a pivotal role in addressing malnutrition and improving outcomes in perioperative and hospitalized patients. Conclusions: Dietary interventions and ONS represent valuable therapeutic tools in IBD management. Future research should prioritize standardized, well-powered clinical trials and personalized nutritional approaches to better define their role within integrated care pathways.

## 1. Introduction

Inflammatory Bowel Diseases (IBD), encompassing Crohn’s Disease (CD) and Ulcerative Colitis (UC), are chronic, immune-mediated disorders characterized by persistent inflammation of the gastrointestinal tract [1,2]. The pathogenesis involves a complex interplay between genetic susceptibility, environmental triggers, immune dysregulation, and alterations in the gut microbiome [3,4]. Among environmental factors, diet has emerged as a critical modulator in both disease onset and progression, with food components potentially influencing intestinal inflammation, barrier function, and microbial composition [5,6,7]. Prospective cohort studies have shown that the development of IBD has increased risk associated with emulsifiers such as carboxymethylcellulose and a high proportion of ultra-processed food (UPF) intake, while good adherence to a diet rich in fruit and vegetables, rich in *n*-3 fatty acids, and low in *n*-6 fatty acids intake appear protective [8,9,10,11]. The European Prospective Investigation into Cancer and Nutrition (EPIC) study found that high consumption of sugar, confectionery, and soft drinks combined with low intake of vegetables and unprocessed seafood was associated with an increased risk of UC development [12]. Similarly, a cohort study demonstrated that early-life diet influences IBD risk, with high fish intake and increased consumption of vegetables and fruits at age 1 associated with lower risk, while sugary drink intake correlated with increased risk [13].

There is substantial evidence that specific dietary patterns can impact general health and IBD outcomes, particularly in the postoperative setting [14,15,16,17]. The quality of diet influences body composition, with both under- and over-nutrition impacting outcomes for patients with IBD [18]. Survey-based studies have assessed dietary perceptions in IBD patients, finding that while only 15% believe diet can trigger IBD onset, nearly 60% think that diet plays a role in disease relapse [19]. As a result, many patients avoid specific foods to prevent flare-ups [20], with studies showing that up to 80% of individuals with IBD already modify their diets in hopes of managing symptoms, often without professional guidance [21,22]. However, this restrictive approach can lead to malnutrition, the prevalence of which in IBD patients ranges from 20% to 85%, varying with disease activity, location, and age [23]. This nutritional deficit stems from multiple factors: reduced food intake due to gastrointestinal symptoms, impaired nutrient absorption from mucosal damage, increased metabolic demands during inflammation, protein-losing enteropathy, and medication–nutrient interactions [24]. The consequences of malnutrition in IBD extend beyond weight loss to include impaired immune function, delayed wound healing, increased infection risk, and compromised response to therapy [25]. In fact, malnourished patients experience greater disease activity with more frequent exacerbations, require hospitalization at higher rates, and face elevated risks of serious infections [26,27].

In recent years, diet has been recognized as both a potential risk factor for IBD development [28] and a therapeutic target for disease management [29]. Most research has focused on CD, with fewer studies examining UC or IBD collectively. These range from exclusive formula-based diets to structured whole-food dietary patterns that aim to modulate inflammation, restore nutritional status, and potentially influence disease course. This creates a compelling opportunity for evidence-based dietary interventions that can address nutritional deficiencies, reduce inflammatory triggers, support mucosal healing, and potentially increase the efficacy of pharmacological treatments [30]. Unlike medications that target specific inflammatory pathways, comprehensive dietary approaches can simultaneously influence multiple disease mechanisms while empowering patients with a non-pharmacological tool to participate in their care. The following sections examine specific dietary strategies with varying levels of evidence supporting their implementation in different IBD scenarios, from active disease to maintenance therapy.

Concurrently, oral nutritional supplementation (ONS) has been recognized as an essential component of nutritional care in IBD, addressing specific deficiencies and supporting overall nutritional adequacy.

**Figure 1:**
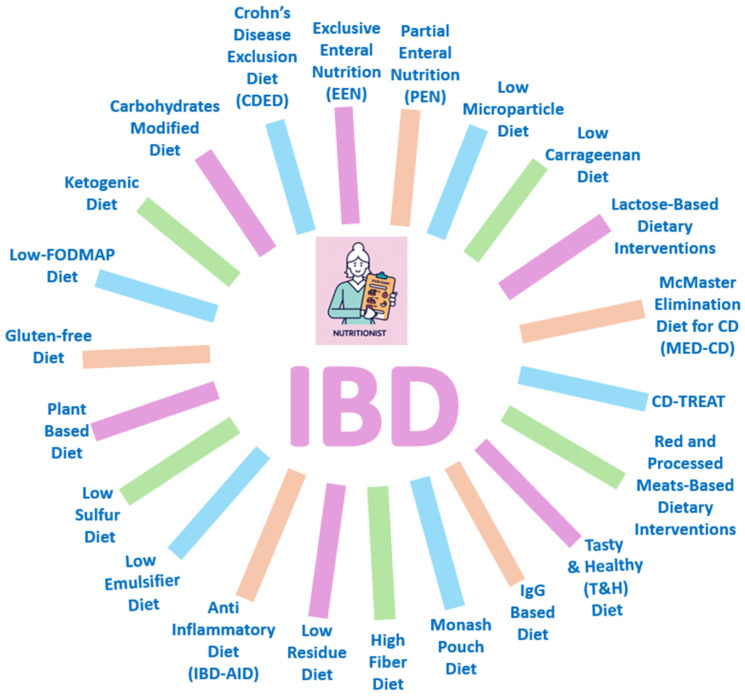
Main Dietary Interventions in Patients with Inflammatory Bowel Disease.

This review aims to provide a comprehensive analysis of the current evidence on dietary interventions and ONS in IBD management, examining various dietary strategies (Figure 1) and discussing their efficacy, proposed mechanisms, practical implementation, and limitations.

A comprehensive literature search was conducted in PubMed, Scopus, and Web of Science to identify relevant studies on dietary strategies and ONS in IBD. The search included the following keywords: *Inflammatory bowel disease* OR *IBD*, *Crohn’s disease* OR *CD*, *Ulcerative colitis* OR *UC*, AND *dietary intervention, medical nutrition therapy, Oral Nutritional Supplementation* OR *ONS*, *Exclusive Enteral Nutrition* OR *EEN*, *Partial Enteral Nutrition* OR *PEN*, *Crohn’s disease exclusion diet* OR *CDED*, *specific carbohydrate diet* OR *SCD*, *Mediterranean diet, low-FODMAP diet, gluten-free diet, plant-based diet, high-fiber diet, low sulfur diet, low emulsifiers diet, ketogenic diet, Anti-IBD diet, nutritional support, malnutrition, anti-inflammatory diet, diet adherence, personalized nutrition* OR *precision nutrition*.

Additional studies were identified through manual searches of reference lists from key review articles and clinical guidelines.

## 2. Dietary Interventions in IBD

### 2.1. Exclusive Enteral Nutrition

Exclusive Enteral Nutrition (EEN) consists of the exclusive intake of a nutritionally complete liquid formula as the sole source of nutrition, typically for a duration of 6–8 weeks, with the exclusion of all other foods and beverages except water [31]. EEN formulas can be categorized as elemental (comprising free amino acids), semi-elemental (containing peptides), or polymeric (based on whole proteins), with the choice tailored to patient-specific needs [32]. While oral administration is the preferred route, enteral feeding via nasogastric or nasojejunal tubes may be necessary when oral intake is inadequate or poorly tolerated. Clinical outcomes in CD appear to be comparable regardless of the route of administration. In children, particularly those who struggle with palatability or volume tolerance, tube feeding is often required to ensure full adherence to the therapeutic regimen.

EEN is well-established as a first-line therapy for inducing remission in mildly active pediatric CD, with international guidelines endorsing its use in this setting [33]. A meta-analysis by Yu et al. reported comparable remission rates between EEN and corticosteroids in children with CD (OR 1.26; 95% CI 0.77–2.05), with additional benefits including improved nutritional status, mucosal healing, and the avoidance of corticosteroid-related adverse effects [34].

In contrast, the use of EEN in adult patients with CD remains limited, largely due to challenges in adherence and acceptability [35].

The elemental diet, one subtype of EEN, delivers all essential nutrients through a highly specialized formula composed of free amino acids, simple carbohydrates, and minimal fat. In the landmark East Anglian Multicenter Controlled Trial [36], Riordan et al. demonstrated that dietary management following remission induced by an elemental diet may be as effective as, or superior to, corticosteroid therapy. In that study, 78 patients in remission after a 14-day elemental diet were randomized to either corticosteroids or a structured food reintroduction protocol. The exclusion diet group showed a significantly longer median remission duration (7.5 vs. 3.8 months) and lower two-year relapse rates (62% vs. 79%, *p* = 0.048), with parallel improvements in inflammatory markers such as plasma albumin, α1-antichymotrypsin, and erythrocyte sedimentation rate.

Similarly, a Japanese randomized controlled trial (RCT) involving 51 CD patients in remission found that those receiving a half elemental diet (50% of daily caloric intake from elemental formula) had a significantly lower relapse rate compared to those on an unrestricted diet (34.6% vs. 64.0%; multivariate HR 0.40, 95% CI 0.16–0.98) [37].

More recently, Diao et al. reported clinical remission at 12 weeks in 73.7% of adult CD patients treated with an amino acid-rich elemental formula, with immunological improvements observed as early as week 4 [38]. Another RCT found that elemental diets were as effective as 6-mercaptopurine in maintaining remission in CD patients [39]. However, the ADORE cohort study showed no significant benefit of combining an elemental diet with anti-TNF therapy, with comparable remission rates at two years (60.9% vs. 56.7%, *p* = 0.98) [40]. Additionally, a study by Kakkadasam Ramaswamy et al. observed that oral polymeric EEN induced clinical remission in 63.6% of adult CD patients at 8 weeks, with higher efficacy among those adhering for at least 6 weeks [41].

Regarding UC, a Cochrane review concluded that there is insufficient evidence to support the effectiveness of nutritional therapy in active disease [42]. Nonetheless, a recent RCT found that EEN combined with intravenous corticosteroids reduced the likelihood of steroid failure in acute severe UC compared to steroids alone (intention-to-treat: 25% vs. 43%, *p* = 0.051; per protocol: 19% vs. 43%, *p* = 0.04) [43].

Despite these findings, the role of EEN in adult patients remains less well-defined. Unlike in the pediatric population, where it is a first-line therapy, in adults it is considered a supportive treatment. While EEN can be effective in inducing remission in adults with mild active CD, it is generally considered inferior to drug treatment (corticosteroids or biologics) and is not recommended as first-line treatment for most adult patients. However, it may be used in selected adult patients who cannot tolerate or refuse drug treatment.

Overall, while EEN is recognized as a first-line induction therapy in pediatric CD, its role in adults remains less defined. In adult patients, EEN is generally considered a supportive measure rather than a primary therapy, typically reserved for those who are unable or unwilling to take medications such as corticosteroids or biologics. Moreover, no clear guidelines exist regarding the optimal formula, though polymeric formulas with low sodium content are generally better tolerated [44]. Further analysis of oral nutritional supplements (ONS) and their specific formulations will be addressed later in this review.

The mechanisms underlying the efficacy of EEN are not yet fully elucidated but are believed to be multifactorial [45]. Proposed mechanisms include the exclusion of dietary antigens, modulation of gut microbiota composition and function, reduction in intestinal permeability, and suppression of pro-inflammatory cytokine production [46]. Recent evidence also points to the modulation of bile acid metabolism and promotion of intestinal epithelial repair as additional contributors to EEN’s anti-inflammatory effects [47].

Despite its clinical potential, EEN faces practical barriers to implementation in adults, including poor palatability, high cost, social limitations, and the burden of dietary restriction, all of which can impair adherence and quality of life [48]. Strategies to improve acceptance include flavor adjustments, psychological support, and stepwise transition protocols [24]. Recent innovations in formula design, including the addition of bioactive compounds and prebiotics, aim to enhance both efficacy and tolerability [49].

### 2.2. Partial Enteral Nutrition

Partial Enteral Nutrition (PEN) has gained attention as an alternative to Exclusive Enteral Nutrition (EEN), particularly for maintenance therapy and in adult patients for whom full adherence to EEN is often difficult [50]. PEN typically provides 35–50% of daily caloric needs through a liquid formula, combined with a conventional or modified diet. This strategy aims to preserve the therapeutic benefits of enteral nutrition while enhancing quality of life and improving long-term adherence.

Both earlier and more recent studies have evaluated PEN as a standalone or adjunct therapy during induction of remission, in combination with unrestricted diets, standard medical treatments, or specific dietary modifications [51,52,53]. While traditional views regarded PEN as less effective than EEN in active CD, growing evidence suggests that PEN may be effective both in inducing and maintaining remission [51,54,55,56,57]. A systematic review by González-Torres et al. analyzed the use of PEN for induction therapy and reported generally high response rates, especially when PEN was paired with restrictive dietary interventions [50]. In a pilot study, Nardone et al. examined PEN as an adjunct to biologic therapy in refractory CD, reporting improved clinical remission and evidence of transmural healing—suggesting a potential synergistic effect [58].

A recent systematic review and meta-analysis by Jatkowska et al. offered a comprehensive evaluation of PEN’s role in CD management [59]. Based on 64 studies, including 11 randomized controlled trials, the authors identified a dose-dependent relationship between PEN and relapse prevention. Higher levels of PEN intake (>35% of total energy requirements) were significantly associated with lower relapse risk. Specifically, intakes of 35–50% were linked to a reduced relapse risk (OR: 0.42; 95% CI: 0.27–0.65), with even greater protection observed at intakes exceeding 50% (OR: 0.27; 95% CI: 0.08–0.88). Additionally, PEN demonstrated comparable nutritional outcomes to EEN in pediatric populations [59].

The mechanisms underlying PEN’s therapeutic effects are thought to mirror those of EEN, involving modulation of the gut microbiota, reduction in dietary antigen exposure, and decreased intestinal inflammation [46,56,60]. However, the continued intake of whole foods in PEN may reduce its efficacy relative to EEN. On the other hand, the inclusion of specific whole food components—such as polyphenols and omega-3 fatty acids—may confer additional anti-inflammatory properties that enhance the overall therapeutic effect [61].

PEN’s principal advantage lies in its greater palatability and tolerability, particularly among adults, leading to better adherence compared to EEN [62]. Notably, when combined with structured exclusion diets such as the Crohn’s Disease Exclusion Diet (CDED), PEN has demonstrated remission induction rates comparable to EEN, especially in pediatric settings. The synergy between PEN and dietary interventions like CDED merits further investigation, as this combination may offer an optimal balance of nutritional adequacy, clinical efficacy, and patient acceptability. The following section will further explore the components and mechanisms of action of the CDED.

### 2.3. Crohn’s Disease Exclusion Diet

The CDED represents a whole-food-based approach to dietary management of CD [52] and it is suggested by ESPEN guidelines in adult patients with mild to moderate active CD or as an alternative to EEN in pediatric patients with mild to moderate CD [63].

Developed by Levine et al., this diet was designed to reduce exposure to dietary components hypothesized to negatively affect the microbiome, intestinal barrier, and immunity. The CDED protocol spans 18 weeks divided into three six-week phases, with decreasing reliance on enteral nutrition as the diet progresses. Phase 1 features mandatory consumption of fish, chicken breast, and eggs, along with permitted foods including rice, cooled potatoes, tomatoes, alliums, and selected oils. Limited amounts of specific fruits and vegetables are allowed. Phase 2 introduces additional proteins (tuna), grains (whole-grain bread, oats), and vegetables (red peppers, beans, peas, root vegetables). Phase 3 (Maintenance) further expands the diet to include more seafood, eggs, cocoa, coffee, grains, some dairy products, and alcohol if tolerated.

The landmark RCT by Levine et al. compared CDED plus PEN to EEN in children with mild-to-moderate CD [52]. The study demonstrated that CDED + PEN was as effective as EEN inducing remission in 6 weeks (75% vs. 70%, *p* = 0.55) but showed superior sustainability at 12 weeks (75.6% vs. 45.1%, *p* = 0.01) due to better dietary adherence. Moreover, both interventions similarly reduced inflammatory markers and favorably altered the fecal microbiome, supporting the biological plausibility of CDED’s therapeutic effects.

Building on these findings, Yanai et al. conducted an open-label pilot trial of CDED + PEN in adults with mild-to-moderate CD [64]. The results showed clinical remission rates of 62% at 6 weeks and 55% at 12 weeks, suggesting that CDED’s efficacy extends to adult populations. More recently, Pasta et al. performed an RCT comparing CDED to a control diet in adult CD patients and reported significantly higher remission rates with CDED at both 12 and 24 weeks [65]. In an open-label, pilot randomized trial involving 44 biologic-naive patients with mild-to-moderate CD, the efficacy of the CDED alone versus CDED + PEN was investigated. By week 6, over 50% of patients in both groups had achieved clinical remission, defined as a Harvey–Bradshaw Index (HBI) score below 5, with no significant difference between the two groups. At 24 weeks, 80% of the patients maintained sustained clinical remission, and 35% also achieved endoscopic remission, defined as a Simple Endoscopic Score for Crohn’s Disease (SES-CD) of 3 or less.

The mechanisms underlying CDED’s efficacy appear multifaceted. By eliminating potential dietary triggers, CDED may reduce exposure to components that compromise intestinal barrier function or promote microbial dysbiosis [66]. Additionally, studies have demonstrated that CDED alters fecal microbial composition, increasing beneficial species such as *Faecalibacterium prausnitzii* while decreasing potentially harmful bacteria, including adherent-invasive *Escherichia coli* [67]. These microbial changes may contribute to reduced intestinal inflammation and improved barrier function.

From a practical perspective, CDED’s whole-food approach offers several advantages over formula-based diets, including improved palatability, social acceptability, and lower cost [68]. However, the diet’s restrictive nature requires detailed nutritional guidance and regular monitoring to ensure nutritional adequacy [69].

### 2.4. Mediterranean Diet

The Mediterranean Diet (MD) has gained considerable attention as a potential therapeutic approach for IBD due to its overall health benefits [70]. Characterized by a high intake of olive oil, fruits, vegetables, legumes, and whole grains; moderate consumption of fish and poultry; and limited red meat and processed foods, the MD provides a rich source of bioactive compounds with potential immunomodulatory effects [71,72,73,74].

Evidence supporting the MD’s benefits in IBD comes from several observational and interventional studies [75]. A prospective cohort study by Godny et al. investigated the impact of the MD on newly diagnosed CD patients and found that higher adherence to the MD was inversely correlated with inflammatory biomarkers and microbial dysbiosis [76]. Similarly, a prospective study by Chicco et al. including 142 IBD patients (84 with UC and 58 with CD) who followed the MD for six months showed significant improvements in nutritional status, liver steatosis, clinical disease activity, and quality of life [77]. Additionally, Marsh et al. conducted a pilot RCT of a modified anti-inflammatory dietary pattern (IBD-MAID), a diet that focuses on eliminating food additives (non-nutritive sweeteners, emulsifiers, carrageenan, maltodextrin, and nitrates) while incorporating Mediterranean Diet principles [78]. They found significant improvements in symptoms, quality of life, and inflammatory markers in CD patients, though not in UC.

In terms of comparative efficacy, Lewis et al. conducted an RCT comparing the Specific Carbohydrate Diet (SCD) to the MD in adults with mild-to-moderate CD [79]. The study found no significant difference between the two diets in terms of clinical remission (SCD 46.5% vs. MD 43.5%, *p* = 0.77), fecal calprotectin reduction (SCD 34.8% vs. MD 30.8%, *p* = 0.83), or C-reactive protein (CRP) levels (SCD 5.4% vs. MD 3.6%, *p* = 0.68). These data suggest that the MD may be as effective as more restrictive dietary approaches while potentially offering greater adherence and sustainability.

MD’s beneficial effects in IBD may stem from several mechanisms. The diet is rich in polyphenols, omega-3 fatty acids, and dietary fiber, all of which have demonstrated anti-inflammatory properties [70]. Additionally, the MD has been shown to promote a diverse and stable gut microbiome, characterized by increased beneficial bacteria and their metabolites, such as short-chain fatty acids (SCFAs) [80].

From a clinical perspective, the MD offers several advantages in IBD management. Compared to more restrictive diets, the MD provides a balanced nutritional profile, greater food variety, and cultural acceptability, potentially enhancing long-term adherence. Furthermore, its established cardiovascular and metabolic benefits address the increased risk of comorbidities observed in the IBD population [81,82]. For these reasons, the American Gastroenterological Association’s (AGA) clinical practice update recommends the MD as a suitable long-term dietary approach for IBD patients, particularly during remission phases [83].

### 2.5. Carbohydrates Modified Diet

The SCD was initially developed by Dr. Sidney Haas in the 1920s for celiac disease management and later popularized by Elaine Gottschall for IBD treatment [84,85]. The SCD is based on the premise that complex carbohydrates, particularly disaccharides and polysaccharides, are poorly absorbed in the inflamed intestine and serve as substrates for pathogenic bacteria, potentially exacerbating inflammation [61]. Accordingly, the diet eliminates grains, starchy vegetables, lactose, sucrose, and processed foods while allowing specific monosaccharides, unprocessed meats, certain fruits and vegetables, and fermented dairy (specifically homemade yogurt) [86].

Evidence supporting the SCD’s efficacy in IBD comprises primarily observational studies and case series [87,88,89,90], in addition to the aforementioned RCT by Lewis et al. [79]. A case series by Kakodkar et al. reported clinical improvement in 33 IBD patients following the SCD, with 42% achieving complete remission [88]. Similarly, a survey-based study by Suskind et al. found that 66% of IBD patients who tried the SCD reported clinical improvement, with many able to reduce or discontinue medications [91]. However, higher-quality evidence from controlled trials has yielded more nuanced results. The N-of-1 trial series evaluated the SCD and its modified version (MSCD) in pediatric patients with IBD, finding no consistent benefit over usual diets [90]. While some individuals showed improvement, overall results did not support a meaningful difference between SCD and MSCD in reducing symptoms or inflammation.

Two other notable studies have investigated the therapeutic potential of carbohydrate modification in CD. Lorenz-Meyer et al. conducted a randomized controlled multicenter trial comparing omega-3 fatty acids and a carbohydrate-reduced diet (84 g/day) against placebo for maintenance of remission in 204 CD patients [92]. While the omega-3 fatty acid intervention showed no benefit over placebo (30% vs. 30% remaining in remission at one year), patients who adhered to the low-carbohydrate diet demonstrated a significant benefit (53% remaining in remission, *p* = 0.023). However, in intention-to-treat analysis, this advantage diminished (40% vs. 30% for placebo), highlighting the critical role of dietary adherence.

In a complementary investigation, Ritchie et al. published in 1987 a controlled multicenter trial comparing an unrestricted sugar, low-fiber diet against a low-sugar, high-unrefined carbohydrate diet in 352 patients with inactive or mildly active CD [93]. After two years of follow-up, no clear differences in clinical outcomes were observed between the two dietary approaches.

The proposed mechanisms underlying the SCD include starving pathogenic bacteria of complex carbohydrate substrates, reducing fermentation and gas production, decreasing intestinal permeability, and modulating the gut microbiome composition [94].

From a practical standpoint, the SCD presents significant challenges in implementation and adherence. The diet’s restrictive nature, complexity, and requirement for extensive food preparation can impact quality of life and contribute to nutritional inadequacies if not properly monitored [95]. A study assessed the nutritional adequacy of the SCD in pediatric patients with IBD, finding overall adequate intake for most nutrients but notable deficiencies in vitamin D and calcium [96]. Consequently, patients undertaking the SCD require comprehensive nutritional guidance, regular follow-up, and potential supplementation to ensure nutritional adequacy.

Despite its popularity among patients, current evidence does not support the SCD as a first-line dietary approach for IBD management. Given its comparable efficacy to less restrictive diets like MD, clinicians should consider factors such as patient preference, adherence capability, and nutritional requirements when discussing the SCD as a treatment option.

### 2.6. Low-FODMAP Diet

The Low-Fermentable Oligosaccharides, Disaccharides, Monosaccharides, And Polyols (FODMAPs) Diet was originally developed for irritable bowel syndrome (IBS) management, but it has gained attention in IBD due to the high prevalence of IBS-like symptoms [97,98,99].

The low-FODMAP is an elimination-rechallenge diet that restricts fermentable oligosaccharides, disaccharides, monosaccharides, and polyols, short-chain carbohydrates that are poorly absorbed in the small intestine [100]. These are excluded from the diet for up to 6 weeks, followed by their gradual reintroduction and by a final personalization phase. This approach may be beneficial to consider in patients with IBD who present with concurrent IBS-like symptoms; however, as no trials have specifically targeted this overlap, there are currently no definitive evidence-based recommendations available. Emerging research in IBS suggests that certain FODMAPs (e.g., fructans, mannitol, GOS) are more likely to cause symptoms, and simplified approaches may be effective, though such data are not yet available for IBD [101].

Low-FODMAP ‘s efficacy in IBD comes primarily from studies focused on functional gastrointestinal symptoms rather than inflammatory activity [102,103,104,105,106,107,108]. A meta-analysis by Zhan et al. evaluated the efficacy of the low-FODMAP in patients with IBD in remission and found significant improvements in gastrointestinal symptoms, including diarrhea, abdominal bloating, pain, fatigue, and nausea [109]. Similarly, a systematic review and meta-analysis by Peng et al. demonstrated that the low-FODMAP effectively alleviated functional gastrointestinal symptoms in IBD patients, particularly those without active disease [104].

An RCT involving 52 patients with IBD who followed a low-FODMAP diet demonstrated a reduction in intestinal symptoms in 52% of patients (compared to 16% of patients on the control diet, *p* = 0.007) [105]. However, no significant difference was found between the two groups in the change in IBS severity scores, contradicting previous findings [110]. Importantly, this study also assessed inflammatory markers and microbiome composition, finding no adverse effects on either parameter during the intervention period.

One of the key challenges in low-FODMAP diet trials is accurately defining the FODMAP threshold that constitutes a “low” intake. This is complicated by individual variability in tolerance, inconsistencies in food composition, and evolving analytical methodologies. While a daily intake of less than 12 g is generally recommended during the restriction phase, there are no universally accepted, gram-specific guidelines for each FODMAP subgroup. Notably, the reported presence of lactose in studies examining IBS symptoms in IBD has been inconsistent, further complicating interpretation [106].

In recognition of the significant clinical problem of persistent diarrhea even in patients with stable disease, the recent MODULATE trial aimed to compare multiple therapeutic approaches for managing diarrhea in patients with stable UC [111]. This pragmatic, multicenter, phase 2/3 RCT was set to evaluate the efficacy of low-FODMAP against pharmacological interventions including amitriptyline, ondansetron, and loperamide. However, its early closure due to COVID-19-related recruitment challenges, leaves the question on how to effectively treat IBS-symptoms in IBD unsolved and highlighting the need for future studies exploring dietary and pharmacological approaches to manage functional symptoms that persist despite adequate control of mucosal inflammation.

The low-FODMAP ‘s mechanism of action in symptom improvement likely involves reduced gas production, decreased intestinal distension, and modulation of gut motility [112]. By limiting rapidly fermentable carbohydrates, the low-FODMAP may reduce bacterial fermentation products that trigger abdominal discomfort, bloating, and altered bowel patterns in susceptible individuals [113].

Moreover, despite its benefits for symptom management, concerns exist regarding the low-FODMAP’s long-term impact on nutritional adequacy and gut microbiota. FODMAPs, particularly oligosaccharides, serve as important prebiotics that support beneficial bacteria in the colon [114]. Their prolonged restriction may lead to reduced microbial diversity and decreased production of beneficial metabolites such as butyrate, although evidence is not conclusive [115].

Consequently, current recommendations emphasize implementing the low-FODMAP as a short-term intervention with a structured reintroduction phase to identify individual triggers while minimizing unnecessary restrictions [116]. In clinical practice, the low-FODMAP should be considered for IBD patients with persistent symptoms despite adequate control of inflammation. Implementation requires guidance from a specialized dietitian [117] and typically follows a three-phase approach including the personalized long-term management [118,119]. This approach allows for symptom improvement while minimizing unnecessary dietary restrictions and potential negative impacts on microbiota diversity.

### 2.7. Anti-Inflammatory Diet

The Inflammatory Bowel Disease Anti-Inflammatory Diet (IBD-AID) was developed as a modification of the SCD, incorporating additional components based on emerging research on diet and inflammation [120]. The IBD-AID is based on multiple synergetic principles: limiting specific carbohydrates such as lactose and refined sugars; incorporating prebiotics, probiotics, and foods that support a healthy microbiota; reducing total and saturated fat intake while increasing omega-3 fatty acid consumption; assessing the diet for food intolerances and nutrient deficiencies; and modifying food texture to facilitate digestion and absorption. The approach follows a progressive, phased strategy based on symptom severity, beginning with soft, pureed foods during disease flares and gradually reintroducing more fibrous, whole foods as symptoms improve and remission is achieved.

Evidence supporting the IBD-AID is still limited. A case series by Olendzki et al. reported that 60% of patients adhering to the IBD-AID experienced good or very good clinical responses, with significant reductions in disease activity indices [120]. A more recent study by Keshteli et al. showed that an anti-inflammatory dietary pattern was effective in preventing subclinical colonic inflammation and modulating the metabolomic profile in UC patients in clinical remission [121].

The proposed mechanisms of action for the IBD-AID include modulation of the gut microbiota, reduction in dietary pro-inflammatory triggers, and incorporation of anti-inflammatory components such as omega-3 fatty acids and polyphenols [122]. However, larger controlled trials are needed to confirm its clinical efficacy and to further elucidate the underlying biological mechanisms.

### 2.8. Plant-Based Diet

Plant-based diets (PBDs), ranging from vegetarian to vegan patterns, have garnered increasing attention for their potential anti-inflammatory properties and favorable effects on the gut microbiota [123,124]. These diets emphasize the consumption of whole plant foods rich in fiber, polyphenols, and antioxidants, while limiting or excluding animal products and processed foods [125].

A comprehensive review by Liu and Day evaluated 23 studies involving 2304 participants to assess PBDs’ efficacy in IBD management [126]. Most studies reported that PBDs were both safe and effective in reducing disease activity and maintaining remission, although several methodological limitations were noted. Notably, a prospective study investigating a semi-vegetarian diet (SVD) demonstrated significant efficacy in preventing CD relapses, with 94% of patients on the SVD maintaining remission compared to only 33% of those following an omnivorous diet [127].

Recent evidence from two large prospective cohort studies provides strong epidemiological support for the benefits of plant-based diets in IBD. Chen et al. analyzed data from the UK Biobank (187,888 participants) and the EPIC cohort (341,539 individuals) across eight European countries [128]. They found that higher adherence to a healthy PBD index was associated with a significantly lower IBD risk (HR 0.75, 95% CI 0.60–0.94 in UK Biobank; HR 0.71, 95% CI 0.59–0.85 in EPIC), while an unhealthy PBD was associated with increased risk (HR 1.48, 95% CI 1.21–1.82 in UK Biobank; HR 1.54, 95% CI 1.30–1.84 in EPIC). Importantly, for disease course and comorbidities, a healthy PBD was associated with reduced risk of IBD-related surgery (HR 0.50, 95% CI 0.30–0.83), while an unhealthy PBD increased this risk (HR 2.12, 95% CI 1.30–3.44). These findings highlight the importance of not just increasing plant-based foods, but ensuring the overall quality of the diet, with potentially stronger benefits for those with genetic predisposition to IBD.

The mechanisms underlying PBDs’ potential benefits include increased fiber intake leading to enhanced SCFA production, reduced exposure to pro-inflammatory components in animal products, and increased consumption of plant-derived anti-inflammatory compounds [129]. Additionally, PBDs may promote a more diverse and stable gut microbiome, contributing to improved intestinal barrier function and immune regulation [125].

While promising evidence for PBDs in IBD management requires confirmation from larger, well-designed clinical trials. Additionally, patients adopting PBDs should receive guidance on ensuring nutritional adequacy, particularly regarding protein, iron, zinc, calcium, vitamin B12, and vitamin D [130].

### 2.9. Low-Sulfur Diet

Sulfur-containing compounds present in the diet can be metabolized by gut bacteria into hydrogen sulfide (H_2_S), which, at elevated concentrations, may disrupt the colonic mucus layer and promote intestinal inflammation [131]. As results, low-sulfur diets have been investigated, particularly in UC, where increased sulfide production has been implicated in disease pathogenesis [132,133].

The 4-SURE diet (4 Strategies to SUlfide-REduction) focuses on reducing dietary sulfur while promoting gut health through other mechanisms. A feasibility study in 28 adults with mild-to-moderately active UC demonstrated that the 4-SURE diet was well-tolerated, with 95% adherence, and it resulted in a clinical response in 46% and endoscopic improvement in 36% of participants [134]. Additionally, the diet led to increased fecal SCFAs and improved food-related quality of life. Given the small sample size, further evaluation is needed in a placebo-controlled trial.

Ongoing research is also investigating whether an Ulcerative Colitis Exclusion Diet (UCED) may serve as an effective treatment option for patients with UC [135]. In an initial study involving adult patients with clinically confirmed endoscopic disease, including 29% who were steroid-refractory and 55% who were biologic-refractory, patients receiving UCED alone exhibited the highest rates of clinical (40%) and endoscopic remission (27%) compared to those receiving fecal transplantation with or without dietary preconditioning of the donor [136].

The proposed mechanisms underlying the benefits of low-sulfur diets include limiting the availability of substrates for sulfate-reducing bacteria, thereby reducing H_2_S production and its associated pro-inflammatory effects [137]. Additional strategies, such as increasing fermentable fiber to stimulate SCFA production and lowering colonic pH, may further suppress the activity of sulfide-producing microbes.

### 2.10. Low-Emulsifier Diet

Food-grade emulsifiers, commonly used in processed foods to improve texture and extend shelf life, have been implicated in promoting intestinal inflammation and disrupting the gut microbiota in preclinical models [138,139,140,141,142]. Their potential role in the pathogenesis of IBD has become increasingly evident in recent years. However, while certain emulsifiers have been associated with IBD development [143], their elimination alone should not be regarded as a definitive therapeutic strategy [6].

A feasibility study by Sandall et al. demonstrated that emulsifier restriction is achievable in people with CD and may improve symptoms and disease control [144]. However, a subsequent RCT by Fitzpatrick et al. found no significant difference in disease activity between high-emulsifier and low-emulfisier diet in CD patients over a four-week period, though both diets improved quality of life and fatigue [145].

Recent evidence from the ADDapt trial, a multicenter, randomized, double-blind, placebo-controlled re-supplementation study involving 154 patients with mild to moderate active CD, has shown that the low-emulfisier diet is an effective therapeutic approach. These findings were presented at the most recent European Crohn and Colitis Congress (ECCO 2025) [146], although the full paper is still pending publication. In this innovative trial design, participants were randomized to either follow the low-emulfisier diet alone or the low-emulfisier diet with emulsifier re-supplementation (control group). The results demonstrated that 49.4% of patients in the low-emulfisier diet group achieved a clinical response (defined as a ≥70-point reduction in the Crohn’s Disease Activity Index, CDAI), compared to only 30.7% in the control group (*p* = 0.019). Moreover, patients adhering to the low-emulfisier diet were more than twice as likely to achieve clinical remission and to experience a >50% reduction in fecal calprotectin levels.

While these findings are promising and suggest a potential benefit of emulsifier avoidance, further research is needed to validate these outcomes. Until more definitive evidence becomes available, a prudent approach may involve minimizing the intake of heavily processed foods as part of a broader, health-promoting dietary pattern, rather than focusing solely on emulsifier exclusion.

### 2.11. Tasty & Healthy Diet

The Tasty & Healthy (T&H) diet has recently emerged as a potential alternative, designed to exclude processed foods, gluten, red meat, and dairy (except plain yogurt) while avoiding mandatory formula-based supplementation [147].

A randomized, controlled, physician-blinded trial (TASTI-MM) evaluated the efficacy and tolerability of the T&H diet compared to EEN in children and young adults with mild-to-moderate CD over an 8-week period [147]. The trial demonstrated that the T&H diet was non-inferior to EEN in inducing clinical remission, as measured by the weighted Pediatric Crohn’s Disease Activity Index (wPCDAI) and CDAI, with no significant differences in biochemical remission markers, including CRP, ESR, and fecal calprotectin levels. Notably, adherence rates were significantly higher in the T&H group (88%) compared to EEN (52%), suggesting that the flexibility of the T&H diet enhances patient compliance. These results were presented at ECCO 2025; however, the full paper has not yet been published.

These findings underscore the potential of whole-food-based dietary strategies as an alternative to traditional formula-based enteral nutrition for induction therapy in CD. Future research should focus on long-term outcomes, microbiome-mediated mechanisms, and the potential role of T&H in maintenance therapy to further define its place in CD management algorithms.

### 2.12. Ketogenic Diet

The ketogenic diet is characterized by severe restriction of carbohydrates (typically 20–50 g per day or less than 5–10% of total energy intake), moderate protein consumption, and significantly increased fat intake (up to 70–90% of total calories) [148]. This macronutrient distribution is thought to force the body to shift from glucose-based metabolism to fat metabolism, producing ketone bodies. Originally developed in the 1920s as a therapeutic approach for drug-resistant epilepsy [149], the ketogenic diet has more recently gained attention for potential applications in various neurological, metabolic, and inflammatory conditions, though its mechanism of action and therapeutic potential vary significantly depending on the specific pathology being addressed [150].

The ketogenic diet’s application in IBD represents an area of significant scientific uncertainty. Preclinical studies have yielded contradictory results: Li et al. demonstrated exacerbation of colitis with increased inflammation and intestinal permeability in mouse models [151], while Kong’s team observed the opposite effect, noting reduced inflammatory cell infiltration and beneficial microbiome alterations [152]. The minimal human evidence consists largely of Norwitz and Soto-Mota’s uncontrolled case series of 10 patients reporting symptom improvement on a carnivore-ketogenic regimen [153]. This contradictory landscape raises substantial concerns about the diet’s safety and efficacy profile in IBD. The carbohydrate restriction inherent to ketogenic diets drastically reduces dietary fiber intake, potentially compromising microbiome diversity and reducing production of SCFAs that maintain intestinal barrier integrity [154]. Furthermore, the diet’s high fat content raises questions about systemic inflammatory effects and cardiovascular implications [155]. Without properly designed RCTs that include objective markers of mucosal healing alongside symptomatic assessment, the ketogenic diet remains an experimental approach that should be considered only with appropriate clinical monitoring and a clear understanding of its unproven status in IBD management.

### 2.13. Gluten-Free Diet

A gluten-free diet involves the complete elimination of gluten, a group of proteins found in wheat, barley, rye, and sometimes oats due to cross-contamination [156]. This dietary approach is primarily indicated for celiac disease [157,158], non-celiac wheat sensitivity [159], and wheat allergy, though it has also gained popularity among individuals with various gastrointestinal disorders seeking symptomatic relief [160]. Many IBD patients adopt gluten-free diets despite limited supporting evidence for this practice. A substantial cross-sectional study involving 1647 IBD patients found approximately one-fifth of IBD patients had attempted gluten elimination, with nearly two-thirds self-reporting symptomatic improvement and over one-third perceiving reduced disease flares [161]. However, these observations must be viewed cautiously as they derive from subjective patient reports rather than objective clinical measurements within controlled trials. The AGA emphasizes there is no consistent evidence supporting routine gluten avoidance in IBD patients [83]. Moreover, perceived benefits from gluten-free diets may instead result from the concurrent reduction in FODMAPs that often accompanies gluten restriction [159,162]. This overlap makes it difficult to attribute symptom improvement specifically to gluten elimination. The substantial nutritional restrictions, increased food costs, and potential social limitations associated with gluten-free diets underscore the importance of recommending this approach only when clinically indicated by confirmed gluten-related disorders rather than as a routine intervention for IBD management.

### 2.14. Low-Residue Diet

The low-residue diet remains a common recommendation for IBD patients despite its limited supporting evidence [163]. This dietary approach restricts fiber and other indigestible components with the goal of reducing stool bulk and frequency, thereby theoretically minimizing mechanical irritation of inflamed or strictured bowel segments. Clinicians typically recommend the LRD during acute disease flares, perioperative periods, or in cases of symptomatic intestinal strictures [164]. The diet eliminates high-fiber foods like whole grains, legumes, nuts, seeds, and certain fruits and vegetables, as well as potentially irritating dairy products and tough meats. However, research examining the efficacy of low-residue diet in IBD management has yielded disappointing results. A prospective study of 70 patients with active CD found no significant differences between those following LRD versus an unrestricted diet regarding symptom control, complications, hospitalizations, surgeries, or nutritional status [165]. The Low Fat/Fiber Limited Exclusion (LOFFLEX) diet was studied a maintenance strategy following remission induction in CD [166]. Woolner et al. evaluated this approach against a traditional elimination diet in 76 treatment episodes, with patients self-selecting either the LOFFLEX (63%) or elimination diet (37%) after achieving remission through enteral nutrition or TPN. The LOFFLEX diet demonstrated slightly superior compliance rates while maintaining comparable efficacy in sustaining remission. At the 24-month mark, approximately 56% of compliant, non-strictured patients on the LOFFLEX diet remained in remission, nearly identical to the 59% observed in the elimination diet group. While encouraging, the retrospective nature of this analysis highlights the need for prospective RCTs to definitively establish the LOFFLEX diet’s role in maintaining CD remission.

Moreover, systematic reviews indicate that other dietary approaches, particularly exclusion diets targeting specific food components, show more promising outcomes than general fiber restriction [167,168]. Importantly, prolonged adherence to low-residue diet raises concerns about adverse impacts on gut health, as dietary fiber serves as essential substrate for beneficial gut bacteria. The resulting reduction in microbial diversity and production of SCFAs may potentially compromise intestinal barrier function and immune regulation [169,170]. Current guidelines, therefore, position low-residue diet as a short-term intervention rather than a long-term management strategy.

### 2.15. High-Fiber Diet

Dietary fiber plays a complex role in IBD management by modulating gut microbiota composition and function, which directly influences intestinal inflammation and barrier integrity [171]. Recent research examining fiber-rich diets across IBD populations reveals promising findings, though research using whole-food approaches remains limited compared to studies of isolated fiber supplements. The effectiveness of high-fiber diets varies significantly between UC and CD and depends heavily on disease activity and individual patient factors.

For UC, emerging evidence supports the beneficial effects of higher fiber intake. Hallert et al. conducted an open multicenter trial where UC patients in remission consumed the equivalent to 20 g of dietary fiber for 12 weeks [172]. The results demonstrated a 36% increase in fecal butyrate concentration at 4 weeks, with significant improvements in gastrointestinal symptoms, particularly abdominal pain, by week 12. Importantly, no patients exhibited signs of colitis relapse during the intervention period.

In a two-period crossover study by James et al., UC patients in remission and healthy controls were randomized to receive either a high resistant starch/wheat bran or a low resistant starch/wheat bran diet [173]. The high-fiber intervention was well-tolerated and tended to normalize gut transit.

Fritsch et al. implemented a more comprehensive dietary approach in their randomized, parallel-group crossover study. UC patients in remission or with mild disease received either a low-fat, high-fiber diet providing only 10% of calories from fat, or an improved Standard American Diet with 35–40% calories from fat but higher quantities of fruits, vegetables, and fiber than typical [174]. Both dietary patterns significantly increased fiber content compared to participants’ baseline diets and improved quality of life. However, only the low-fat, high-fiber diet resulted in a significant reduction in serum amyloid A and showed a trend toward decreased CRP, suggesting additional anti-inflammatory benefits beyond increased fiber intake alone. These benefits appear to be mediated through increased production of SCFAs, particularly butyrate.

For CD, the evidence is more limited. Traditional concerns about fiber exacerbating stricturing disease have led to widespread fiber restriction. However, recent research challenges this paradigm. In an RCT, Brotherton et al. demonstrated that a high-fiber diet incorporating wheat bran was not only feasible but also beneficial for patients with CD [175]. Participants consuming wheat bran reported significant improvements in health-related quality of life (*p* = 0.028) and gastrointestinal function (*p* = 0.008) compared to controls. Notably, this intervention did not trigger adverse effects or disease exacerbation. This finding gains additional support from a subsequent observational study that analyzed data from the Crohn’s and Colitis Foundation of America Partners Internet cohort [176]. Among 1130 CD patients in remission followed for six months, those in the highest quartile of fiber consumption demonstrated a 42% reduction in disease flare risk compared to those with the lowest intake (adjusted OR 0.58; 95% CI 0.37–0.90). Perhaps most tellingly, patients who reported not avoiding high-fiber foods were approximately 40% less likely to experience a disease flare than those who restricted fiber (adjusted OR 0.59; 95% CI 0.43–0.81). A meta-analysis by Serrano Fernandez et al. found that high-fiber intake was not associated with worsening symptoms in non-stricturing CD [177]. This suggests that the quality and preparation of fiber may be more important than total fiber content.

The AGA now recommends incorporating fiber-rich foods as part of a MD for most IBD patients in remission, with texture modifications for those with stricturing disease [83]. This approach maintains the beneficial effects of fiber on gut microbiota while minimizing mechanical irritation. Importantly, fiber restriction should be considered a short-term intervention during acute flares rather than a long-term management strategy, as prolonged restriction may adversely affect gut health and microbiome diversity.

### 2.16. McMaster Elimination Diet for Crohn’s Disease

The McMaster Elimination Diet for Crohn’s Disease (MED-CD) is a whole-food diet that excludes processed foods, emulsifiers, thickeners, gluten, and dairy while permitting unprocessed meats, fruits, vegetables, gluten-free grains, legumes, nuts, and select beverages.

In a pilot study of 17 patients with mild-to-moderate CD, 13 responded to an initial 2-week enteral nutrition phase and continued to the 12-week MED-CD phase [178]. The intervention demonstrated high adherence (84.6% at week 6, 67% at week 14), with 38.5% of patients maintaining clinical remission and 46.2% achieving endoscopic response by week 14. Inflammatory markers significantly improved, with better outcomes observed in patients with ileal/ileocolonic disease versus isolated colonic disease, and in never-smokers versus current/former smokers.

Larger controlled trials are needed to definitively establish the efficacy of MED-CD.

### 2.17. IgG-Based Diet

The concept of customizing dietary exclusions based on individual immunological responses has been explored in two notable studies examining food-specific IgG antibody-guided elimination diets in IBD [179,180]. In Bentz et al., a double-blind cross-over study with 40 patients with CD found that eliminating foods with high IgG antibody levels significantly reduced stool frequency by 11% compared to a sham diet while also improving abdominal pain and general well-being. Similarly, Rajendran and Kumar’s pilot study with 29 patients with CD showed that excluding each patient’s four most IgG4-reactive foods for four weeks resulted in symptomatic improvement in 90% of participants, with significant reductions in modified CDAI (171 to 97.5, *p* = 0.0001) and inflammatory markers.

Beyond IBD, IgG-based elimination diets have been investigated across several other conditions, most extensively in IBS but also in migraine disorders, autoimmune conditions including rheumatoid arthritis and psoriasis, and weight management programs [181,182,183,184,185]. Despite some positive results, major medical organizations have expressed caution about their widespread application, noting that IgG antibodies may simply indicate exposure rather than pathology.

### 2.18. CD-TREAT Diet

Svolos et al. developed CD-TREAT (Crohn’s Disease treatment-with-EATing) diet, a whole-food-based dietary approach designed to mimic the nutritional composition of EEN, as a more palatable alternative for managing CD [186]. CD-TREAT was meticulously designed to match EEN’s macronutrient and micronutrient composition, including protein, carbohydrate, and fat ratios, while mimicking other nutritional characteristics such as fiber content, fatty acid profile, and specific food component exclusions. In a three-part study, they first demonstrated in healthy volunteers that CD-TREAT induced similar fecal microbiome and metabolome alterations to EEN, including comparable changes in bacterial diversity, SCFAs, and pH. These findings were then validated in HLA-B27 transgenic rats with gut inflammation, where both diets similarly reduced ileitis severity and altered the gut microbiome. Finally, in a pilot trial with five children with active CD, CD-TREAT led to clinical response in 80%, clinical remission in 60%, and significant reductions in fecal calprotectin (mean decrease of 55%). Participants also rated CD-TREAT as more palatable and easier to follow than EEN, suggesting its potential as a more acceptable dietary alternative with comparable therapeutic mechanisms.

Despite these encouraging results, several limitations must be acknowledged. The small sample size of the clinical pilot study necessitates larger, adequately powered trials to confirm efficacy. Furthermore, the lack of a direct head-to-head comparison with EEN in the clinical setting makes it difficult to definitively assess equivalence. Additional studies are also needed to evaluate long-term adherence, nutritional adequacy, and the maintenance of remission over time. Nevertheless, CD-TREAT represents a promising innovation in the dietary management of Crohn’s disease and warrants further clinical investigation through well-designed randomized controlled trials.

### 2.19. Lactose-Based Dietary Interventions

The potential role of dietary allergens, particularly milk proteins, in triggering or exacerbating UC has been a subject of clinical investigation for nearly a century. In a landmark 1965 paper, Wright and Truelove conducted one of the earliest controlled trials examining dietary interventions in UC [187]. They designed a stratified randomized study comparing three dietary approaches: a milk-free diet, a combined gluten-free and milk-free diet, and a control “dummy” diet. Their interest stemmed from earlier observations that patients who became symptom-free on milk-free diets frequently relapsed when milk was reintroduced. This controlled approach was necessary due to the variable and unpredictable nature of UC remissions and relapses. Nearly five decades later, Strisciuglio et al. (2013) conducted a similar randomized trial specifically assessing the role of cow’s milk protein elimination in pediatric UC [188]. Their study of 29 children found that 86.2% achieved remission with standard therapy regardless of diet group, and relapse rates after one year were virtually identical between the elimination diet (53.8%) and free diet (53.3%) groups. The researchers concluded that cow’s milk protein elimination offers no benefit for non-sensitized children with UC.

### 2.20. Red and Processed Meats-Based Dietary Interventions

In an RCT, Albenberg et al. investigated whether reducing consumption of red and processed meats would decrease the risk of symptomatic relapse in patients with CD in remission [189]. The study, known as the Food and Crohn’s Disease Exacerbation Study (FACES), recruited 213 participants from an internet-based cohort who were in clinical remission. Participants were randomized to either a high-meat diet (minimum 2 servings of red/processed meat weekly) or a low-meat diet (maximum 1 serving monthly) for 49 weeks.

Participant adherence was excellent, with the high-meat group consuming the required amount of meat in 98.5% of observed weeks, compared to only 18.8% of weeks in the low-meat group. Despite this clear dietary distinction, there were no significant differences in time to relapse between the groups. Clinical relapse occurred in 62% of high-meat group participants versus 42% in the low-meat group, but this difference was not statistically significant (*p* = 0.61). The researchers concluded that reducing red and processed meat consumption does not appear to reduce the risk of CD relapse in patients in clinical remission.

### 2.21. Low Microparticle Diet

Two studies investigated the therapeutic potential of reducing dietary microparticle intake in patients with active CD. Microparticles are non-biological, bacteria-sized particles (primarily titanium dioxide and silicates) found in processed foods, pharmaceuticals, and toothpaste that accumulate in intestinal lymphoid tissue and may potentially influence inflammatory responses.

In the initial pilot study by Lomer et al., 20 patients with active, corticosteroid-treated CD were randomized to either a low microparticle diet or a control diet for four months [190]. Patients on the low microparticle diet showed progressive improvement in CDAI scores from 392 at baseline to 145 at four months, with 70% achieving remission. In contrast, the control group showed no improvement, with CDAI scores remaining around 300 and no patients achieving remission. These striking results led to a subsequent multi-center trial by the same research group, sought to validate these findings with a larger cohort and more rigorous design [191]. This study enrolled 83 patients with active CD in a 2 × 2 factorial design examining both microparticle intake (low vs. normal) and calcium intake (low vs. normal). All patients received a standard reducing course of prednisolone. Unlike the pilot study, this larger trial found no difference in remission rates, clinical response, quality of life, or inflammatory markers between patients on low versus normal microparticle diets over the 16-week intervention or 36-week follow-up period.

The authors propose several possible explanations for these contradictory findings. The smaller pilot study may have had a type II error with an unusually steroid-resistant control group. Additionally, baseline disease severity was lower in the follow-up study, potentially masking benefits that might only be apparent in more severe disease. Methodological differences in diet implementation may also have contributed, as the initial study used a more stringent elimination of all processed foods.

### 2.22. Low Carrageenan Diet

A randomized, double-blind, placebo-controlled trial conducted by Bhattacharyya et al. investigated whether carrageenan, a common food additive with known inflammatory properties in experimental models, contributes to disease relapse in patients with UC in remission [192].

The study enrolled 12 participants who followed a carrageenan-free diet and were randomized to receive either capsules containing 200 mg of carrageenan daily or similar-appearing placebo capsules. All participants were carefully instructed to avoid carrageenan-containing processed foods such as ice cream, yogurt, chocolate milk, processed meats, and other items where carrageenan is commonly used as a thickening agent.

The results showed a statistically significant difference in relapse rates between the two groups. Three patients in the carrageenan-supplemented group experienced disease relapse during the 12-month follow-up period, while none in the placebo group relapsed (*p* = 0.046). Inflammatory biomarkers, including interleukin-6 (*p* = 0.02) and fecal calprotectin (*p* = 0.06), increased significantly in the carrageenan-exposed group but not in the placebo group.

These findings suggest that dietary carrageenan may contribute to inflammation and disease relapse in UC patients, providing a potentially modifiable environmental factor in disease management, although larger trials are needed to confirm these findings.

### 2.23. Diet in Special Situations: Pouchitis Management

Patients who undergo colectomy or proctocolectomy for inflammatory bowel disease face unique nutritional challenges related to altered gut anatomy and physiology. Dietary guidance is frequently provided to these patients in an effort to improve stool consistency and volume, reduce urgency and frequency, prevent dehydration, and mitigate complications such as pouchitis [193]. Despite the routine nature of such dietary counseling in clinical practice, high-quality evidence supporting specific nutritional recommendations remains surprisingly limited.

Several dietary approaches have shown promise in managing post-colectomy symptoms. Reducing FODMAP intake has been shown to decrease ileal and ileostomy output [194]. More recently, the Monash Pouch Diet, specifically designed for patients with an ileal pouch anal anastomosis (IPAA), combines elements of the low-FODMAP approach with additional modifications tailored to pouch physiology. The MPD incorporates five key principles: increased oligosaccharide intake (6–8 g/day), reduced total protein intake (75–100 g/day), limited sulfur-containing proteins from animal sources, restricted osmotically active carbohydrates, and minimized intake of sulfur-containing food preservatives. In a 6-week pilot trial with 12 patients, the MPD demonstrated excellent tolerability in 75% of participants and high acceptability in 81%. All six symptomatic patients achieved clinical remission (*p* = 0.03), although one patient withdrew after developing a partial small bowel obstruction [195].

Pouchitis is a well-known complication in patients with UC who undergo total colectomy and J-pouch creation, affecting up to 60% of these patients and is notoriously difficult to manage [196,197]. Diet may play an important role in its management [198]. Epidemiological data point to potential protective effects of certain dietary patterns. An adequate fruit consumption has been linked to a lower risk of developing pouchitis [199], potentially due to beneficial effects on the pouch microbiome or through direct anti-inflammatory mechanisms. Another study on 153 patients followed after pouch surgery observed that adherence to the MD was associated with decreased fecal calprotectin and CRP levels and was inversely associated with the onset of pouchitis (*p* = 0.17) [200].

A study involving dietary questionnaires given to patients with a pouch revealed that those with pouchitis consumed less fruit and vegetables compared to those with a healthy pouch [201].

A prospective study evaluated the effectiveness of an exclusive elemental diet in patients with pouchitis. Although the study included only seven patients, results after 28 days showed significant improvement in symptoms and stool frequency (Pouchitis Disease Activity Index (PDAI) symptom score decreased from 4 to 1, *p* = 0.039; stool frequency from a median of 12 to 6, *p* = 0.028) [202].

An interventional pilot study examined whether a fiber-adapted CDED is effective for the treatment of acute pouchitis [203]. Among the eight patients who completed the protocol and were supplemented with 2000 international units of vitamin D and 1000 mg calcium per day, clinical remission was achieved in 66.7% at week 6 and maintained in 46.7% of participants at week 24. Stool frequency, bowel urgency, and serum CRP levels significantly decreased by week 12, and diet-induced remission was negatively associated with a severe endoscopic PDAI score. However, 46% of participants withdrew from the study due to worsening symptoms or bowel obstruction, highlighting that dietary interventions are not without risks and may have limited clinical applicability.

Importantly, patients with an ileal pouch often report numerous perceived food intolerances [201]. As such, individualized dietary counseling may be necessary to reduce food-related anxiety and improve quality of life.

In clinical practice, a fiber-restricted MD may support both prevention and management of pouchitis [95]. Additional measures could be patient-tailored, such as avoiding high-FODMAP foods to reduce pouch output and adjusting meal timing to minimize nocturnal symptoms.

### 2.24. Diet in Special Situations: Perioperative Nutritional Management

Despite advancements in biologic therapies and new treatment options, surgery remains a viable treatment for both CD and UC patients [204]. Patients requiring surgical intervention frequently present with active disease and compromised nutritional status, which significantly impacts postoperative outcomes, including increased risks of infectious complications, anastomotic leakage, and mortality [205]. While high-quality RCTs in this specific area remain limited, the ECCO consensus provides valuable guidance for the perioperative nutritional management of IBD patients, a crucial yet often overlooked component of comprehensive surgical care [206].

The Enhanced Recovery After Surgery (ERAS) protocol represents a multidisciplinary, evidence-based approach to perioperative care designed to optimize patient outcomes by minimizing physiological stress and accelerating recovery. Although originally developed for colorectal surgery, ERAS principles have been successfully extended to various surgical specialties, including IBD procedures [207,208,209]. The protocol emphasizes the critical importance of identifying and addressing malnutrition during the perioperative period, with prehabilitation strategies aimed at improving lean body mass before surgery to mitigate the catabolic impact of surgical stress [210].

Key nutritional components of ERAS in IBD surgery include avoiding prolonged preoperative fasting, early postoperative oral feeding, integrating nutrition into overall patient management, optimizing metabolic control (particularly glucose regulation), reducing factors that exacerbate stress-related catabolism, and promoting early mobilization to support protein synthesis and muscle function.

Evidence strongly supports routine nutritional assessment before planned IBD surgery [211]. When malnutrition is identified, evidence strongly supports delaying elective surgery for 7–14 days to implement intensive nutritional rehabilitation, as this approach significantly reduces postoperative complications and improves surgical outcomes [63]. This recommendation stems from studies demonstrating that “severe” nutritional risk—defined as weight loss > 10–15% within six months, BMI < 18.5 kg/m^2^, Nutritional Risk Screening (NRS) score > 5, or serum albumin < 30 g/L without hepatic or renal dysfunction—substantially increases postoperative complications. Notably, both undernutrition and obesity function as independent risk factors, with weight loss > 10% in the preceding six months being particularly predictive of adverse outcomes [212]. In CD specifically, preoperative optimization through EEN for 4–6 weeks has shown promising results in reducing CRP levels, shortening surgery duration, and decreasing complication rates, though comparable evidence for UC remains limited [207]. A meta-analysis examining 1111 CD patients found that those receiving nutritional support (enteral or parenteral) had a significantly lower risk of postoperative complications (20% vs. 61.3%, OR = 0.26, 95% CI: 0.07–0.99, *p* < 0.001), with enteral nutrition specifically associated with reduced postoperative morbidity (21.9% vs. 73.2%, OR = 0.09, 95% CI 0.06–0.13, *p* < 0.001) [208]. Additionally, a prospective study demonstrated that CD patients who received preoperative EEN experienced significant improvements in HBI scores, CRP levels, and albumin concentrations compared to controls [209]. Interestingly, EEN also reduced microbiota diversity, specifically with a decrease in Enterobacteriaceae [210].

For patients unable to meet nutritional requirements through regular diet alone, ONS is recommended during the perioperative period [211]. Numerous studies have demonstrated significant advantages from nutritional supplementation, particularly regarding the reduction in infectious complications, length of hospital stay, and overall costs [212,213]. The enteral route should always be prioritized when feasible, with parenteral nutrition reserved for cases where enteral feeding is impossible or contraindicated.

While preoperative nutritional optimization is valuable, it should never delay urgent surgical intervention in emergency situations [214]. Postoperatively, early oral feeding according to enhanced recovery pathways is recommended, as this approach does not compromise anastomotic healing and facilitates more rapid recovery and shorter hospital stays.

### 2.25. Diet in Special Situations: Stenosis

Patients with IBD who develop luminal stenosis require special dietary considerations to minimize the risk of obstructive complications. While symptomatic obstruction is relatively uncommon compared to the overall prevalence of stricturing disease, careful dietary management remains essential. Traditionally, patients with stricturing disease have been advised to avoid foods with high fiber content, particularly those that maintain their form during intestinal transit, such as raw fruits and vegetables with peels, nuts, seeds, and fibrous vegetables [171,215]. While patients with radiologically confirmed but asymptomatic strictures are typically advised to consume a diet low in insoluble fiber, this practice, although logical, lacks strong supporting evidence [216]. When symptoms are present, dietary adjustments, such as transitioning to a soft-texture or fluid-based, nutrient-rich diet, may be necessary. In fact, the physical properties and preparation of fiber-rich foods are more critical than fiber content alone. The ESPEN now recommends focusing on “adapted textures” rather than complete fiber elimination [63]. This approach allows patients with stricturing disease to consume soluble fibers and even some insoluble fibers that have been properly modified through cooking, blending, or pureeing [83]. Recent evidence suggests that many patients with strictures can tolerate a wider variety of foods when properly prepared [216]. A recent pilot study examined 20 patients with stricturing CD, comparing a high-protein, low-fiber diet alone versus the same diet plus essential amino acids and sodium butyrate supplementation for 12 months [217]. The supplemented group showed significantly improved body composition (particularly skeletal muscle index), reduced inflammation markers, and lower surgery rates (10% versus 40% in controls). For severe or symptomatic strictures, EEN may temporarily reduce inflammation and improve luminal diameter. In fact, a prospective observational study examined the effectiveness of EEN therapy for inflammatory bowel strictures [218]. Sixty-five patients with CD and inflammatory bowel strictures were treated with 12 weeks of EEN: results showed that 50 patients (84.7%) completed the full treatment, while 9 patients (15.3%) required surgery due to progressive bowel obstruction. Among all participants, 81.4% achieved symptomatic remission, 53.8% achieved radiologic remission, and 64.6% achieved clinical remission. For those who completed the therapy, significant improvements were observed in inflammatory markers, nutritional parameters, and bowel measurements. Most notably, the average luminal cross-sectional area at the stricture site increased dramatically by approximately 331% at week 12 compared to baseline. Similarly, in a retrospective analysis of 82 patients with stricturing CD, Marafini et al. evaluated whether implementing a periodic 24 h liquid diet every 10–14 days improved clinical outcomes [219]. Their findings revealed no significant difference in sub-occlusive episodes between patients receiving the liquid diet intervention (27%) versus standard therapy alone (20%). Surgical intervention rates were similarly unaffected (24.3% vs. 15.5%). Parenteral nutrition represents a vital nutritional intervention when enteral feeding methods prove inadequate, particularly in case of strictures, when confronting bowel obstructions that prevent successful feeding tube placement beyond the blockage point or after failed tube placement attempts [63]. Despite promising results from individual studies, the evidence supporting specific dietary approaches for managing stricturing disease remains limited by small sample sizes, lack of RCTs, and significant heterogeneity in study designs and patient populations. The degree of dietary restriction should be individualized based on the severity and location of strictures, current symptoms, and nutritional status [220]. Importantly, unnecessarily restrictive diets should be avoided to prevent malnutrition, and patients should be encouraged to gradually reintroduce foods in modified forms with guidance from a registered dietitian. Regular reassessment of dietary tolerance is essential as stricture severity may change with disease management.

Table 1 summarizes the main dietary interventions in IBD management.

## 3. Oral Nutritional Supplementation in IBD

Malnutrition represents a pervasive and clinically consequential complication in IBD, affecting up to 85% of patients with CD and 60% with UC [230]. This nutritional compromise reflects the multifactorial interplay between inflammatory burden, compromised nutrient intake, and impaired intestinal absorption [23]. To counteract these deleterious nutritional trajectories, ONS has emerged as a pivotal therapeutic intervention in contemporary IBD management [231]. These specialized formulations deliver concentrated macro- and micronutrients in highly bioavailable forms, strategically designed to supplement conventional dietary intake when nutrient requirements cannot be adequately met through food alone. Available in diverse delivery systems, ranging from energy-dense liquids to specialized powders, ONS provides targeted nutritional intervention while circumventing the need for more invasive feeding modalities. Particularly during periods of disease exacerbation, when inflammatory cytokines drive catabolic processes and symptom burden limits oral intake, ONS serves as a critical nutritional bridge, potentially interrupting the bidirectional relationship between malnutrition and inflammation that can otherwise accelerate disease progression. As the paradigm of IBD management evolves beyond symptomatic control toward comprehensive care, ONS increasingly represents not merely an adjunctive strategy but a fundamental component of optimized disease management with significant implications for clinical outcomes, therapeutic response, and patient quality of life.

Regulations for ONS vary by country. In Europe, they are categorized as Foods for Special Medical Purposes (FSMPs), which means they are specially formulated for managing malnutrition in patients and require medical supervision. ONS plays a crucial role in addressing these nutritional challenges, particularly during active disease phases when dietary intake may be compromised.

ONS includes a broad range of products designed to supplement regular diet with macro- and micronutrients in a concentrated, easily digestible form [232]. ONSs are in various formats (liquid, powder, pudding, pre-thickened), volumes, flavors (or no flavor: e.g., LH Viola©), and nutritional compositions. In IBD, commonly used ONS categories include high-calorie supplements to address energy deficits when providing ≥1.5 kcal/mL (ESPEN guidelines) or ≥1.2 kcal/mL (Italian legislation); high-protein supplements to compensate for increased protein requirements and losses, when providing ≥20% of energy from protein; low-residue formulations to minimize gastrointestinal symptoms during flares; and disease-specific formulations containing anti-inflammatory components such as transforming growth factor-beta (TGF-β) or specific fatty acids [233]. ONSs may also contain various compounds with anti-inflammatory properties, such as omega-3 fatty acids, vitamins E and C, carotenoids, polyphenols, and minerals like selenium and zinc, which offer health benefits [31]. Typically, manufacturers define their products’ intended use and target malnutrition type, as there is no comprehensive legislation specifically defining ONS nutritional composition requirements [234].

In clinical practice, ONSs are typically used to provide energy- and protein-dense nutrition in a low-volume format. For adults with IBD, energy supplementation up to 600 kcal/day from ONSs can be achieved without displacing normal food intake [63]. ESPEN guidelines recommend ONSs when spontaneous intake is inadequate, highlighting their value in preoperative nutrition, during recovery, and for long-term maintenance of nutritional goals.

The benefits of ONS in malnourished IBD patients extend beyond simple weight gain. A systematic review and meta-analysis by Cawood et al. demonstrated that high-calorie, high-protein ONS significantly reduced complications in malnourished patients, including lower infection rates, improved wound healing, and reduced healthcare utilization [235].

A recently published analysis of FSMPs available on the Italian market by Madini et al. [236] identified 21 products potentially suitable for use during active IBD. These included formulations low in fat (hypolipidic or lipid-free), options with medium-chain triglycerides (MCTs) to support fat absorption, and amino acid-based supplements designed to minimize gastrointestinal stress. Notably, 91% of these products were fiber-free, and all products identified were also lactose-free, further supporting tolerability.

In remission, ONSs can help maintain nutritional adequacy in patients with persistent low intake, malabsorption, or ongoing nutrient losses. The analysis by Madini et al. found 32 products deemed appropriate for this phase, most of which were hypercaloric (59–81%) and hyperproteic (approximately 69%) [236]. These supplements offered customizable options, including fiber-free formulas for early remission and fiber-enriched formulations (up to ~5 g/portion) for patients reintroducing dietary fiber. The inclusion of fermentable fibers, such as inulin, fructooligosaccharides (FOS), and galactooligosaccharides (GOS), may also benefit microbiota composition.

Only a limited number of ONSs contain immune-modulating nutrients like arginine, glutamine, omega-3 fatty acids, or β-hydroxy-β-methylbutyrate (β-HMB), which have been suggested to support mucosal healing, muscle mass preservation, and anti-inflammatory pathways. However, these types of formulations are not recommended by guidelines due to lack of high quality data supporting their use [63]. Multiple Cochrane meta-analyses have shown that elemental, semi-elemental, and polymeric enteral nutrition formulas are similarly effective in inducing clinical remission in active CD, with no significant differences in outcomes [237,238,239,240]. Variations in protein composition [232,240] as well as specialized nutritional products, including those enriched with growth factors, reduced emulsifiers, or based on oligomeric formulations [233] also appear to have little to no influence on therapeutic success. Notably, the inclusion of additives that have been hypothesized to contribute to CD pathogenesis, such as carrageenan or modified starches, did not adversely affect clinical outcomes [42].

Although some studies have indicated that very low-fat or low long-chain triglyceride (LCT) formulas may be associated with slightly higher remission rates [31], this does not exclude the potential use of high medium-chain triglyceride (MCT) formulations in certain clinical contexts.

This suggests that formula selection should be based on individual patient factors, including tolerance, cost, and specific nutritional requirements, rather than presumed superior efficacy of any particular formulation.

Despite their clinical utility, the absence of standard compositional requirements specific to IBD represents a barrier to optimal ONS design. Current products are largely positioned for general malnutrition or disease-related needs rather than IBD-specific phenotypes. Furthermore, high osmolarity, a feature present in the majority of ONSs, may contribute to gastrointestinal intolerance in sensitive patients if not consumed gradually and at appropriate temperatures.

Table 2 summarizes classifications and characteristics of ONS used in IBD management.

## 4. Future Directions and Research Needs

### 4.1. Personalized Nutrition Approaches

The future of nutritional therapy in IBD increasingly lies at the intersection of precision medicine and advanced multi-omics technologies. The heterogeneity of IBD in terms of genetic background, microbiome composition, and environmental factors suggests that standardized dietary interventions may yield suboptimal results, necessitating personalized approaches for maximal therapeutic efficacy.

Future research should focus on identifying biomarkers and patient characteristics that predict response to specific dietary interventions, enabling tailored recommendations.

Several factors may inform personalized nutrition approaches in IBD. Genetic factors, including polymorphisms in genes involved in nutrient metabolism, immune function, and intestinal barrier maintenance, may influence dietary response [234]. For example, variants in genes encoding pattern recognition receptors might affect responses to specific dietary components through altered microbe-host interactions [241].

An interesting yet evolving area of research is the role of the microbiome in IBD, particularly how diet can influence its composition and, in turn, affect disease symptoms and progression [242]. Consequently, microbiome characteristics represent another potential basis for dietary personalization [243]. Baseline microbiome composition, including the presence of specific bacterial species or functional capacities, may predict responses to dietary interventions [244]. Additionally, monitoring changes in microbiome composition during diet therapy could guide adjustments to optimize beneficial shifts and enhance therapeutic outcomes [245]. However, much remains to be understood about the precise mechanisms through which these changes in the microbiome impact IBD, especially in cases where patients experience overlapping DGBIs [246,247].

Metabolomic profiles, reflecting both host and microbial metabolism, offer insights into individual responses to dietary components [248,249]. Integration of metabolomic data with dietary interventions could identify specific metabolic signatures associated with positive or negative responses, informing personalized recommendations.

Disease phenotype characteristics, including location, behavior, and inflammatory patterns, likely influence dietary response. Developing phenotype-specific dietary algorithms could enhance efficacy by matching interventions to relevant pathophysiological mechanisms [250]. For example, patients with predominantly fibro-stenosing disease might benefit from different dietary approaches than those with primarily inflammatory presentations.

Emerging technologies, including artificial intelligence and machine learning, may facilitate the integration of these complex data sources to generate personalized dietary recommendations [251]. These approaches could process multiple patient-specific variables to predict optimal dietary patterns and guide clinical decision-making.

### 4.2. Standardization of Research Methodologies

Diet trials face unique challenges compared to drug trials. Unlike drug-naïve patients, all IBD patients already have established dietary habits, food preferences, and belief systems that influence compliance and potentially affect study endpoints. Foods contain multiple nutrients and are challenging to control precisely in a research setting. Bench-to-bedside translation of dietary components has significant interpretive limitations, as in vitro models typically use extracted or synthetic components rather than whole foods with complex nutrient compositions. Furthermore, diet interventions are inherently difficult to blind and control in human trials.

Progress in dietary therapy for IBD depends on improving the consistency and quality of research methodologies to facilitate cross-study comparisons [252]. Dietary assessment in clinical research is inherently complex, often affected by measurement errors, recall bias, social desirability, and changes in participant behavior during data collection [253,254,255]. The primary methods, including food diaries, 24 h recalls, and food frequency questionnaires, each have limitations. Selection should be guided by the study’s objectives and population characteristics. For microbiome-focused research, documenting dietary intake for at least two days alongside sample collection provides insights into short-term interactions, though longer tracking may be needed to reflect habitual intake. Nutritional biomarkers offer objective alternatives but currently cover a limited range of dietary components and have quantification challenges.

Systematic reviews of food-based interventions in IBD have revealed considerable variation in trial design and inconsistent outcome reporting. Notably, 46% of trials targeting inflammation lacked primary endpoints measuring inflammatory activity [227]. To enhance comparability, future studies should adopt standardized core outcome sets encompassing clinical assessments, inflammatory biomarkers (e.g., fecal calprotectin, CRP), endoscopic results, patient-reported outcomes (e.g., quality of life, fatigue), and nutritional status indicators. Reporting on dietary adherence, absent in over 40% of trials, also requires more consistent and rigorous methodology.

The choice of control groups is another key methodological factor. Vague comparators such as “usual diet” or “standard care” vary widely and hinder interpretation. Instead, trials should use clearly defined control diets or active comparators to accurately assess the effects of dietary interventions. There is also a need to validate dietary assessment tools specifically in IBD populations. Combining self-reported intake with objective measures, such as biomarkers or digital tools, can improve data accuracy [256]. Furthermore, reporting detailed dietary composition can help isolate effective dietary components [257].

Long-term follow-up is essential to evaluate the durability and adherence of dietary interventions. Many current studies assess outcomes over only 6–12 weeks, limiting understanding of sustained effects. Extending follow-up to 12 months or more would provide more clinically relevant data [258]. Finally, multicenter collaboration using standardized protocols can address limitations in sample size and improve generalizability, while accounting for geographic and cultural differences in diet and care delivery [252].

## 5. Conclusions

Dietary interventions and ONS should not be viewed as mutually exclusive strategies but rather as complementary tools within the nutritional management of IBD. While dietary interventions aim to modulate disease activity through whole-food-based approaches that reduce inflammation, restore gut barrier integrity, and promote a healthy microbiome, ONS primarily address nutritional deficiencies and support adequate caloric and protein intake—especially in patients with active disease, reduced appetite, or increased metabolic demands.

The evidence landscape continues to evolve, with robust support for exclusive enteral nutrition in pediatric CD, promising data for the CDED with PEN across age groups, and increasing recognition of the MD as a sustainable long-term approach. Targeted nutritional supplementation through ONS effectively addresses the multifaceted malnutrition prevalent in this patient population. The complexity of nutritional management in IBD underscores the necessity for dedicated nutrition specialists within multidisciplinary IBD care teams, as nutritional optimization impacts not only disease activity but also functional capacity, therapeutic response, and psychological well-being.

Despite these advances, critical knowledge gaps persist regarding optimal dietary formulations, patient-specific approaches, and synergistic integration with pharmacological therapies. Future research imperatives include methodologically rigorous RCTs with standardized endpoints, development of precision nutrition algorithms based on integrated multi-omics profiling, and engineering of novel nutritional formulations that target specific pathophysiological mechanisms in IBD. With appropriate prioritization, nutritional therapy may evolve from an adjunctive support measure to a cornerstone of precision IBD management, offering new hope for patients navigating the challenges of these complex, chronic conditions.

## Figures and Tables

**Table 1:** Summary of Main Dietary Interventions in Inflammatory Bowel Disease Management.

Dietary Approach	Description	Disease Application	Level of Evidence	Primary Intent	Duration	Key Outcomes	Potential Limitations
Formula-based Interventions							
Exclusive Enteral Nutrition (EEN)	Complete liquid formula as sole nutrition source for defined period	CD (primarily pediatric) [221]	High (pediatric); Moderate (adult)	Induction	6–8 weeks	Clinical remission rates comparable to corticosteroids in pediatric CD; mucosal healing; favorable microbiome changes	Poor palatability; social restrictions; adherence issues in adults
Partial Enteral Nutrition (PEN)	Liquid formula providing 35–50% of calories with conventional diet	CD [51,222]	Moderate	Maintenance	Variable	Dose-dependent prevention of relapse; better long-term adherence than EEN	Limited efficacy as monotherapy without dietary modifications
Whole-food Based Interventions							
Crohn’s Disease Exclusion Diet (CDED)	Three-phase diet excluding dietary triggers, often with PEN	CD (both pediatric and adults) [52,64,65,223]	High (pediatric)/moderate (adults)	Induction; Maintenance	6–24 weeks	Efficacy comparable to EEN in pediatric and adult CD with better adherence	Requires detailed nutritional guidance and monitoring
Mediterranean Diet (MD)	High plant foods, olive oil; moderate fish; limited red meat	CD; UC [79,224,225]	Moderate	Maintenance	Long-term	Similar efficacy to SCD in RCT; improved clinical markers; cardiovascular benefits	Limited evidence for induction of remission
Specific Carbohydrate Diet	Eliminates complex carbohydrates and processed foods	CD [79,226,227,228]	Low-Moderate	Adjunctive; Symptom control	Long-term	Not superior to MD in controlled trials; patient-reported benefits	Highly restrictive; complex implementation; nutritional adequacy concerns
Low-FODMAP Diet	Restricts fermentable carbohydrates with phased reintroduction	IBD with IBS symptoms [102,103,105,108,110,229]	Moderate (symptoms)	Symptom control	2–6 weeks elimination; 6–8 weeks reintroduction; personalization long-term	Effective for functional GI symptoms in quiescent IBD	Not targeting underlying inflammation; concerns about microbiota effects
IBD Anti-Inflammatory Diet	Modified SCD with prebiotics/probiotics and omega-3 emphasis	CD; UC [78,121]	Low	Adjunctive	Variable	Limited evidence from controlled trials	Implementation complexity
Plant-Based Diets	Emphasis on whole plant foods with limited/excluded animal products; ranges from semi-vegetarian to vegan patterns	CD; UC [126,127]	Moderate (epidemiological); Low (interventional)	Maintenance; Risk reduction	Long-term	Prospective cohort evidence for reduced IBD risk and surgery with Healthy plant patterns; improved inflammatory markers	Need for nutritional monitoring for protein, iron, B12, zinc, vitamin D; limited interventional evidence
Ketogenic Diet	Very high fat (70–90%), very low carbohydrate diet inducing ketosis	CD; UC [147]	Very Low	Experimental	Variable	Contradictory preclinical evidence; limited human data from small uncontrolled case series	Drastic fiber reduction affecting microbiome diversity; high saturated fat potentially pro-inflammatory; limited SCFA production; experimental status with minimal clinical evidence
Targeted Exclusion Diets							
Low-Sulfur Diet (4-SURE)	Reduces dietary sulfur to decrease hydrogen sulfide production	UC [134]	Low	Induction	8 weeks	Improvement in colonic mucus layer; reduced hydrogen sulfide toxicity	Limited evidence; complex implementation
Low-Emulsifier Diet	Eliminates food additives in processed foods	CD [140]	Low-Moderate	Adjunctive	2–12 weeks	Emerging clinical trial evidence for symptom improvement	Difficult implementation without broader dietary changes
Tasty & Healthy Diet (T&H)	Excludes processed foods, gluten, red meat, dairy	CD [141]	Moderate (emerging)	Induction	8 weeks	Non-inferior to EEN with significantly better adherence	Limited published evidence
Gluten-Free Diet	Elimination of all gluten-containing foods	IBD [155].	Low	Symptom control	Long-term	Patient-reported symptom improvement	No consistent evidence for IBD without celiac disease
Low-Residue Diet	Restriction of dietary fiber and indigestible components	CD [159]	Low	Symptom control	Short-term	Short-term reduction in mechanical irritation	No improvement in disease outcomes; potential adverse microbiome impact
High-Fiber Diet	Increased fiber consumption with appropriate preparation	UC; CD without strictures [166,168,169,171]	Moderate (UC); Low (CD)	Maintenance	Long-term	Increased SCFA production; improved microbiome diversity	May pose a challenge in stricturing disease
Diet in Special Situations							
Monash Pouch Diet	Modified MD with potential FODMAP restrictions	Pouch [189]	Low	Prevention; Treatment	Long-term	Reduction in inflammation markers; decreased stool frequency	High withdrawal rate in studies; requires individualization

Abbreviations: CD: Crohn’s Disease; UC: Ulcerative Colitis; IBD: Inflammatory Bowel Disease; EEN: Exclusive Enteral Nutrition; PEN: Partial Enteral Nutrition; CDED: Crohn’s Disease Exclusion Diet; FODMAP: Fermentable Oligosaccharides, Disaccharides, Monosaccharides, And Polyols; SCD: Specific Carbohydrate Diet; SCFA: Short-Chain Fatty Acids; RCT: Randomized Controlled Trial; IBS: Irritable Bowel Syndrome; MD: Mediterranean Diet. Level of Evidence: High: Multiple well-designed RCTs or systematic reviews/meta-analyses; Moderate: Limited RCTs, well-designed observational studies; Low: Case series, uncontrolled studies, expert opinion.

**Table 2:** Classification and Characteristics of Oral Nutritional Supplements Used in IBD Management.

ONS Category	Composition	Common Commercial Examples	Clinical Applications in IBD	Advantages	Limitations	Level of Evidence
Elemental Formulas	Free amino acids, minimal fat content (primarily MCTs), simple carbohydrates, vitamins, minerals	Vivonex, Elemental 028, Neocate, E028 Extra	Primary therapy for active CD induction of remission; Used in severe malabsorption; Post-surgical nutrition	Highly digestible and absorbable; Reduced antigenic load; May provide bowel rest	Poor palatability; High cost; Poor compliance; Requires medical supervision	High (CD)
Semi-Elemental/Peptide-Based Formulas	Peptides (hydrolyzed proteins), moderate fat content with MCTs, oligosaccharides, vitamins, minerals	Peptamen, Perative, Vital, Crucial, Peptide 1.5	Moderate malabsorption states; Alternative when elemental formulas not tolerated	Better palatability than elemental; Lower cost than elemental; Good digestibility	Less effective than elemental in some studies; Higher allergenicity than elemental	Moderate
Polymeric Formulas	Whole proteins, balanced fat profile including LCTs, complex carbohydrates, fiber options, vitamins, minerals	Ensure, Boost, Fortisip, Fresubin, Nutrison	Supplemental nutrition in IBD; Prevention of malnutrition in remission phases; Maintenance therapy	Improved palatability; Lower cost; Better compliance; Available in multiple flavors and formats; generally recommended by guidelines	May not be tolerated in severe disease	Moderate (CD); Limited (UC)
Disease-Specific Formulations	Specialized nutrient profiles with anti-inflammatory components (TGF-β, specific fatty acids, glutamine, antioxidants)	Impact Advanced Recovery, Modulen IBD, Alicalm, LH Viola	Targeted therapy for inflammatory modulation; Maintenance therapy	Potential for dual nutritional and disease-modifying effects; Some evidence for maintenance therapy	Higher cost; Limited availability; Newer formulations with evolving evidence base	Low to Moderate
Immunomodulating Formulas	Enhanced levels of omega-3 fatty acids, glutamine, arginine, nucleotides, and antioxidants	Impact, Immun-Aid, Intestamin	Perioperative nutrition in IBD; Adjunctive therapy during flares	Potential to influence disease activity beyond nutritional repletion	Limited IBD-specific evidence; Higher cost; Variable clinical response	Limited

Abbreviations: CD: Crohn’s Disease; IBD: Inflammatory Bowel Disease; LCTs: Long-Chain Triglycerides; MCTs: Medium-Chain Triglycerides; ONS: Oral Nutritional Supplements; RCT: Randomized Controlled Trial; TGF-β: Transforming Growth Factor-beta; UC: Ulcerative Colitis. Levels of Evidence: High: Evidence from multiple well-designed randomized controlled trials or systematic reviews/meta-analyses showing consistent results; Moderate: Evidence from limited RCTs, well-designed observational studies, or controlled trials with some methodological limitations; Low: Evidence primarily from case series, uncontrolled studies, or expert opinion with limited clinical data; Limited: Very few studies available with significant methodological limitations or inconsistent findings.

## References

[1] Le Berre C., Honap S., Peyrin-Biroulet L. (2023). Ulcerative Colitis. Lancet Lond. Engl..

[2] Dolinger M., Torres J., Vermeire S. (2024). Crohn’s Disease. Lancet.

[3] Piovani D., Danese S., Peyrin-Biroulet L., Nikolopoulos G.K., Lytras T., Bonovas S. (2019). Environmental Risk Factors for Inflammatory Bowel Diseases: An Umbrella Review of Meta-Analyses. Gastroenterology.

[4] Barberio B., Facchin S., Patuzzi I., Ford A.C., Massimi D., Valle G., Sattin E., Simionati B., Bertazzo E., Zingone F. (2022). A Specific Microbiota Signature Is Associated to Various Degrees of Ulcerative Colitis as Assessed by a Machine Learning Approach. Gut Microbes.

[5] Jabłońska B., Mrowiec S. (2023). Nutritional Status and Its Detection in Patients with Inflammatory Bowel Diseases. Nutrients.

[6] Halmos E.P., Godny L., Vanderstappen J., Sarbagili-Shabat C., Svolos V. (2024). Role of Diet in Prevention versus Treatment of Crohn’s Disease and Ulcerative Colitis. Frontline Gastroenterol..

[7] Wood J.A., Halmos E.P., Taylor K.M., Gibson P.R. (2021). The Role of Epidemiological Evidence from Prospective Population Studies in Shaping Dietary Approaches to Therapy in Crohn’s Disease. Mol. Nutr. Food Res..

[8] Deng M., Dan L., Ye S., Chen X., Fu T., Wang X., Chen J. (2023). Higher Dietary Fibre Intake Is Associated with Lower Risk of Inflammatory Bowel Disease: Prospective Cohort Study. Aliment. Pharmacol. Ther..

[9] Jantchou P., Morois S., Clavel-Chapelon F., Boutron-Ruault M.-C., Carbonnel F. (2010). Animal Protein Intake and Risk of Inflammatory Bowel Disease: The E3N Prospective Study. Am. J. Gastroenterol..

[10] Khalili H., Håkansson N., Chan S.S., Chen Y., Lochhead P., Ludvigsson J.F., Chan A.T., Hart A.R., Olén O., Wolk A. (2020). Adherence to a Mediterranean Diet Is Associated with a Lower Risk of Later-Onset Crohn’s Disease: Results from Two Large Prospective Cohort Studies. Gut.

[11] Narula N., Wong E.C.L., Dehghan M., Mente A., Rangarajan S., Lanas F., Lopez-Jaramillo P., Rohatgi P., Lakshmi P.V.M., Varma R.P. (2021). Association of Ultra-Processed Food Intake with Risk of Inflammatory Bowel Disease: Prospective Cohort Study. BMJ.

[12] Racine A., Carbonnel F., Chan S.S.M., Hart A.R., Bueno-de-Mesquita H.B., Oldenburg B., van Schaik F.D.M., Tjønneland A., Olsen A., Dahm C.C. (2016). Dietary Patterns and Risk of Inflammatory Bowel Disease in Europe: Results from the EPIC Study. Inflamm. Bowel Dis..

[13] Guo A., Ludvigsson J., Brantsæter A.L., Klingberg S., Östensson M., Størdal K., Mårild K. (2024). Early-Life Diet and Risk of Inflammatory Bowel Disease: A Pooled Study in Two Scandinavian Birth Cohorts. Gut.

[14] Michels K.B., Schulze M.B. (2005). Can Dietary Patterns Help Us Detect Diet-Disease Associations?. Nutr. Res. Rev..

[15] Molendijk M., Molero P., Sánchez-Pedreño F.O., Van der Does W., Martínez-González M.A. (2018). Diet Quality and Depression Risk: A Systematic Review and Dose-Response Meta-Analysis of Prospective Studies. J. Affect. Disord..

[16] Vitale M., Costabile G., Testa R., D’Abbronzo G., Nettore I.C., Macchia P.E., Giacco R. (2024). Ultra-Processed Foods and Human Health: A Systematic Review and Meta-Analysis of Prospective Cohort Studies. Adv. Nutr..

[17] Yamamoto T., Lightner A.L., Spinelli A., Kotze P.G. (2020). Perioperative Management of Ileocecal Crohn’s Disease in the Current Era. Expert Rev. Gastroenterol. Hepatol..

[18] Livingstone K.M., Milte C., Bowe S.J., Duckham R.L., Ward J., Keske M.A., McEvoy M., Brayner B., Abbott G. (2022). Associations Between Three Diet Quality Indices, Genetic Risk and Body Composition: A Prospective Cohort Study. Clin. Nutr. Edinb. Scotl..

[19] Verdina M., Seibold F., Grandmaison G., Michetti P., Barras-Moret A.-C., Liu K., Vaucher J., Staudenmann D. (2023). Survey of Dietary Beliefs and Habits of Inflammatory Bowel Disease Patients. Clin. Nutr. ESPEN.

[20] Godala M., Gaszyńska E., Durko Ł., Małecka-Wojciesko E. (2023). Dietary Behaviors and Beliefs in Patients with Inflammatory Bowel Disease. J. Clin. Med..

[21] Day A.S., Yao C.K., Costello S.P., Andrews J.M., Bryant R.V. (2021). Food Avoidance, Restrictive Eating Behaviour and Association with Quality of Life in Adults with Inflammatory Bowel Disease: A Systematic Scoping Review. Appetite.

[22] Riva A., Arienti G., Zuin G., Spini L., Calia M., Biondi A., Nacinovich R., Cavanna A.E. (2024). Risk Factors for the Development of Eating Disorders in Adolescents with Early-Onset Inflammatory Bowel Diseases. Nutrients.

[23] Liu J., Ge X., Ouyang C., Wang D., Zhang X., Liang J., Zhu W., Cao Q. (2022). Prevalence of Malnutrition, Its Risk Factors, and the Use of Nutrition Support in Patients with Inflammatory Bowel Disease. Inflamm. Bowel Dis..

[24] Bezzio C., Brinch D., Ribaldone D.G., Cappello M., Ruzzon N., Vernero M., Scalvini D., Loy L., Donghi S., Ciminnisi S. (2024). Prevalence, Risk Factors and Association with Clinical Outcomes of Malnutrition and Sarcopenia in Inflammatory Bowel Disease: A Prospective Study. Nutrients.

[25] Shao T., Verma H.K., Pande B., Costanzo V., Ye W., Cai Y., Bhaskar L.V.K.S. (2021). Physical Activity and Nutritional Influence on Immune Function: An Important Strategy to Improve Immunity and Health Status. Front. Physiol..

[26] Yerushalmy-Feler A., Ben-Tov A., Weintraub Y., Amir A., Galai T., Moran-Lev H., Cohen S. (2018). High and Low Body Mass Index May Predict Severe Disease Course in Children with Inflammatory Bowel Disease. Scand. J. Gastroenterol..

[27] Spooren C.E.G.M., Wintjens D.S.J., de Jong M.J., van der Meulen-de Jong A.E., Romberg-Camps M.J., Becx M.C., Maljaars J.P., van Bodegraven A.A., Mahmmod N., Markus T. (2019). Risk of Impaired Nutritional Status and Flare Occurrence in IBD Outpatients. Dig. Liver Dis. Off. J. Ital. Soc. Gastroenterol. Ital. Assoc. Study Liver.

[28] Bancil A.S., Sandall A.M., Rossi M., Chassaing B., Lindsay J.O., Whelan K. (2021). Food Additive Emulsifiers and Their Impact on Gut Microbiome, Permeability, and Inflammation: Mechanistic Insights in Inflammatory Bowel Disease. J. Crohns Colitis.

[29] Reznikov E.A., Suskind D.L. (2023). Current Nutritional Therapies in Inflammatory Bowel Disease: Improving Clinical Remission Rates and Sustainability of Long-Term Dietary Therapies. Nutrients.

[30] Bertin L., Crepaldi M., Zanconato M., Lorenzon G., Maniero D., De Barba C., Bonazzi E., Facchin S., Scarpa M., Ruffolo C. (2024). Refractory Crohn’s Disease: Perspectives, Unmet Needs and Innovations. Clin. Exp. Gastroenterol..

[31] Mitrev N., Huang H., Hannah B., Kariyawasam V.C. (2021). Review of Exclusive Enteral Therapy in Adult Crohn’s Disease. BMJ Open Gastroenterol..

[32] Di Caro S., Fragkos K.C., Keetarut K., Koo H.F., Sebepos-Rogers G., Saravanapavan H., Barragry J., Rogers J., Mehta S.J., Rahman F. (2019). Enteral Nutrition in Adult Crohn’s Disease: Toward a Paradigm Shift. Nutrients.

[33] Day A.S., Lopez R.N. (2015). Exclusive Enteral Nutrition in Children with Crohn’s Disease. World J. Gastroenterol. WJG.

[34] Yu Y., Chen K.-C., Chen J. (2019). Exclusive Enteral Nutrition versus Corticosteroids for Treatment of Pediatric Crohn’s Disease: A Meta-Analysis. World J. Pediatr. WJP.

[35] Tay S., Chan P.W.W., Wei S.C., Tan M., Hilmi I.N., Leong J.W.H., Tan Y.Y., Lim C.T., Ling K.L., Tey T.T. (2024). P579 Dietary Beliefs, Barriers, and Acceptability of Diet in IBD Patients—A Multi-Centre Survey from the Asia-Pacific Region. J. Crohns Colitis.

[36] Riordan A.M., Hunter J.O., Cowan R.E., Crampton J.R., Davidson A.R., Dickinson R.J., Dronfield M.W., Fellows I.W., Hishon S., Kerrigan G.N. (1993). Treatment of Active Crohn’s Disease by Exclusion Diet: East Anglian Multicentre Controlled Trial. Lancet Lond. Engl..

[37] Takagi S., Utsunomiya K., Kuriyama S., Yokoyama H., Takahashi S., Iwabuchi M., Takahashi H., Takahashi S., Kinouchi Y., Hiwatashi N. (2006). Effectiveness of an ‘Half Elemental Diet’ as Maintenance Therapy for Crohn’s Disease: A Randomized-Controlled Trial. Aliment. Pharmacol. Ther..

[38] Diao N., Liu X., Lin M., Yang Q., Li B., Tang J., Ding N., Gao X., Chao K. (2023). Exclusive Enteral Nutrition Orchestrates Immunological Balances as Early as Week 4 in Adult Patients of Crohn’s Disease: A Pilot, Open-Lable Study. Nutrients.

[39] Hanai H., Iida T., Takeuchi K., Arai H., Arai O., Abe J., Tanaka T., Maruyama Y., Ikeya K., Sugimoto K. (2012). Nutritional Therapy versus 6-Mercaptopurine as Maintenance Therapy in Patients with Crohn’s Disease. Dig. Liver Dis. Off. J. Ital. Soc. Gastroenterol. Ital. Assoc. Study Liver.

[40] Hirai F., Ishida T., Takeshima F., Yamamoto S., Yoshikawa I., Ashizuka S., Inatsu H., Mitsuyama K., Sou S., Iwakiri R. (2019). Effect of a Concomitant Elemental Diet with Maintenance Anti-Tumor Necrosis Factor-α Antibody Therapy in Patients with Crohn’s Disease: A Multicenter, Prospective Cohort Study. J. Gastroenterol. Hepatol..

[41] Ramaswamy P.K., Gold Coast Inflammatory Bowel Diseases Research Group (2022). Exclusive Enteral Nutrition with Oral Polymeric Diet Helps in Inducing Clinical and Biochemical Remission in Adults with Active Crohn’s Disease. JPEN J. Parenter. Enteral Nutr..

[42] Limketkai B.N., Iheozor-Ejiofor Z., Gjuladin-Hellon T., Parian A., Matarese L.E., Bracewell K., MacDonald J.K., Gordon M., Mullin G.E. (2019). Dietary Interventions for Induction and Maintenance of Remission in Inflammatory Bowel Disease. Cochrane Database Syst. Rev..

[43] Sahu P., Kedia S., Vuyyuru S.K., Bajaj A., Markandey M., Singh N., Singh M., Kante B., Kumar P., Ranjan M. (2021). Randomised Clinical Trial: Exclusive Enteral Nutrition versus Standard of Care for Acute Severe Ulcerative Colitis. Aliment. Pharmacol. Ther..

[44] Gea Cabrera A., Sanz-Lorente M., Sanz-Valero J., López-Pintor E. (2019). Compliance and Adherence to Enteral Nutrition Treatment in Adults: A Systematic Review. Nutrients.

[45] Melton S.L., Taylor K.M., Gibson P.R., Halmos E.P. (2023). Review Article: Mechanisms Underlying the Effectiveness of Exclusive Enteral Nutrition in Crohn’s Disease. Aliment. Pharmacol. Ther..

[46] Geesala R., Gongloor P., Recharla N., Shi X.-Z. (2024). Mechanisms of Action of Exclusive Enteral Nutrition and Other Nutritional Therapies in Crohn’s Disease. Nutrients.

[47] Bai S.H., Chandnani A., Cao S. (2024). Bile Acids in Inflammatory Bowel Disease: From Pathophysiology to Treatment. Biomedicines.

[48] Mutsekwa R.N., Edwards J.T., Angus R.L. (2021). Exclusive Enteral Nutrition in the Management of Crohn’s Disease: A Qualitative Exploration of Experiences, Challenges and Enablers in Adult Patients. J. Hum. Nutr. Diet. Off. J. Br. Diet. Assoc..

[49] Lunken G.R., Tsai K., Schick A., Lisko D.J., Cook L., Vallance B.A., Jacobson K. (2021). Prebiotic Enriched Exclusive Enteral Nutrition Suppresses Colitis via Gut Microbiome Modulation and Expansion of Anti-Inflammatory T Cells in a Mouse Model of Colitis. Cell. Mol. Gastroenterol. Hepatol..

[50] González-Torres L., Moreno-Álvarez A., Fernández-Lorenzo A.E., Leis R., Solar-Boga A. (2022). The Role of Partial Enteral Nutrition for Induction of Remission in Crohn’s Disease: A Systematic Review of Controlled Trials. Nutrients.

[51] Johnson T., Macdonald S., Hill S.M., Thomas A., Murphy M.S. (2006). Treatment of Active Crohn’s Disease in Children Using Partial Enteral Nutrition with Liquid Formula: A Randomised Controlled Trial. Gut.

[52] Levine A., Wine E., Assa A., Sigall Boneh R., Shaoul R., Kori M., Cohen S., Peleg S., Shamaly H., On A. (2019). Crohn’s Disease Exclusion Diet Plus Partial Enteral Nutrition Induces Sustained Remission in a Randomized Controlled Trial. Gastroenterology.

[53] Hisamatsu T., Kunisaki R., Nakamura S., Tsujikawa T., Hirai F., Nakase H., Watanabe K., Yokoyama K., Nagahori M., Kanai T. (2018). Effect of Elemental Diet Combined with Infliximab Dose Escalation in Patients with Crohn’s Disease with Loss of Response to Infliximab: CERISIER Trial. Intest. Res..

[54] Wall C.L., Gearry R.B., Day A.S. (2018). Treatment of Active Crohn’s Disease with Exclusive and Partial Enteral Nutrition: A Pilot Study in Adults. Inflamm. Intest. Dis..

[55] Yang H., Feng R., Li T., Xu S., Hao X., Qiu Y., Chen M. (2020). Systematic Review with Meta-Analysis of Partial Enteral Nutrition for the Maintenance of Remission in Crohn’s Disease. Nutr. Res..

[56] Brückner A., Werkstetter K.J., Frivolt K., Shokry E., Ahmed M., Metwaly A., Marques J.G., Uhl O., Krohn K., Hajji M. (2020). Partial Enteral Nutrition Has No Benefit on Bone Health but Improves Growth in Paediatric Patients with Quiescent or Mild Crohn’s Disease. Clin. Nutr..

[57] Keetarut K., Kikuchi H., King B., Richards N., Lomer M., Fragkos K., Patel P.S. (2021). Perceived Acceptability of Partial Enteral Nutrition (PEN) Using Oral Nutritional Supplement Drinks in Adolescent and Adult Crohn’s Disease Outpatients: A Feasibility Study. Clin. Nutr. ESPEN.

[58] Nardone O.M., Calabrese G., La Mantia A., Testa A., Rispo A., Alfonsi L., Pasanisi F., Castiglione F. (2024). Effectiveness of Partial Enteral Nutrition as Add-On to Biologics in Patients with Refractory and Difficult-to-Treat Crohn’s Disease: A Pilot Study. Crohns Colitis 360.

[59] Jatkowska A., White B., Gkikas K., Seenan J.P., MacDonald J., Gerasimidis K. (2024). Partial Enteral Nutrition in the Management of Crohn’s Disease: A Systematic Review and Meta-Analysis. J. Crohns Colitis.

[60] Starz E., Wzorek K., Folwarski M., Kaźmierczak-Siedlecka K., Stachowska L., Przewłócka K., Stachowska E., Skonieczna-Żydecka K. (2021). The Modification of the Gut Microbiota via Selected Specific Diets in Patients with Crohn’s Disease. Nutrients.

[61] Fiorindi C., Russo E., Balocchini L., Amedei A., Giudici F. (2022). Inflammatory Bowel Disease and Customized Nutritional Intervention Focusing on Gut Microbiome Balance. Nutrients.

[62] Russell E.E., Day A.S., Dimitroff C., Trakman G.L., Silva H., Bryant R.V., Purcell L., Yao C.K., Landorf E., Fitzpatrick J.A. (2024). Practical Application of the Crohn’s Disease Exclusion Diet as Therapy in an Adult Australian Population. J. Gastroenterol. Hepatol..

[63] Bischoff S.C., Bager P., Escher J., Forbes A., Hébuterne X., Hvas C.L., Joly F., Klek S., Krznaric Z., Ockenga J. (2023). ESPEN Guideline on Clinical Nutrition in Inflammatory Bowel Disease. Clin. Nutr..

[64] Yanai H., Levine A., Hirsch A., Boneh R.S., Kopylov U., Eran H.B., Cohen N.A., Ron Y., Goren I., Leibovitzh H. (2022). The Crohn’s Disease Exclusion Diet for Induction and Maintenance of Remission in Adults with Mild-to-Moderate Crohn’s Disease (CDED-AD): An Open-Label, Pilot, Randomised Trial. Lancet Gastroenterol. Hepatol..

[65] Pasta A., Formisano E., Calabrese F., Apollonio M., Demarzo M.G., Marabotto E., Furnari M., Giannini E.G., Pisciotta L., Bodini G. (2025). The Use of the Crohn’s Disease Exclusion Diet (CDED) in Adults with Crohn’s Disease: A Randomized Controlled Trial. Eur. J. Clin. Investig..

[66] Núñez-Sánchez M.A., Melgar S., O’Donoghue K., Martínez-Sánchez M.A., Fernández-Ruiz V.E., Ferrer-Gómez M., Ruiz-Alcaraz A.J., Ramos-Molina B. (2022). Crohn’s Disease, Host–Microbiota Interactions, and Immunonutrition: Dietary Strategies Targeting Gut Microbiome as Novel Therapeutic Approaches. Int. J. Mol. Sci..

[67] Van Limbergen J., Dunn K., Wine E., Sigall Boneh R., Bielawski J., Levine A. (2020). OP22 Crohn’s Disease Exclusion Diet Reduces Bacterial Dysbiosis Towards Healthy Controls in Paediatric Crohn’s Disease. J. Crohns Colitis.

[68] Sigall Boneh R., Park S., Soledad Arcucci M., Herrador-López M., Sarbagili-Shabat C., Kolonimos N., Wierdsma N., Chen M., Hershkovitz E., Wine E. (2024). Cultural Perspectives on the Efficacy and Adoption of the Crohn’s Disease Exclusion Diet Across Diverse Ethnicities: A Case-Based Overview. Nutrients.

[69] Saibeni S., Zanetti M., Bezzio C., Pironi L., Armuzzi A., Riso S., Caprioli F., Lezo A., Macaluso F.S., Pugliese D. (2023). Nutritional Care at Centres Managing Patients with Inflammatory Bowel Disease: A Nationwide Survey in Italy. Dig. Liver Dis. Off. J. Ital. Soc. Gastroenterol. Ital. Assoc. Study Liver.

[70] Deleu S., Becherucci G., Godny L., Mentella M.C., Petito V., Scaldaferri F. (2024). The Key Nutrients in the Mediterranean Diet and Their Effects in Inflammatory Bowel Disease: A Narrative Review. Nutrients.

[71] Dominguez L.J., Di Bella G., Veronese N., Barbagallo M. (2021). Impact of Mediterranean Diet on Chronic Non-Communicable Diseases and Longevity. Nutrients.

[72] Mazza E., Ferro Y., Pujia R., Mare R., Maurotti S., Montalcini T., Pujia A. (2021). Mediterranean Diet In Healthy Aging. J. Nutr. Health Aging.

[73] Kiani A.K., Medori M.C., Bonetti G., Aquilanti B., Velluti V., Matera G., Iaconelli A., Stuppia L., Connelly S.T., Herbst K.L. (2022). Modern Vision of the Mediterranean Diet. J. Prev. Med. Hyg..

[74] Mentella M.C., Scaldaferri F., Ricci C., Gasbarrini A., Miggiano G.A.D. (2019). Cancer and Mediterranean Diet: A Review. Nutrients.

[75] Marlow G., Ellett S., Ferguson I.R., Zhu S., Karunasinghe N., Jesuthasan A.C., Han D.Y., Fraser A.G., Ferguson L.R. (2013). Transcriptomics to Study the Effect of a Mediterranean-Inspired Diet on Inflammation in Crohn’s Disease Patients. Hum. Genomics.

[76] Godny L., Elial-Fatal S., Arrouasse J., Sharar Fischler T., Reshef L., Kutukov Y., Cohen S., Pfeffer-Gik T., Barkan R., Shakhman S. (2025). Mechanistic Implications of the Mediterranean Diet in Patients With Newly Diagnosed Crohn’s Disease: Multi-Omic Results from a Prospective Cohort. Gastroenterology.

[77] Chicco F., Magrì S., Cingolani A., Paduano D., Pesenti M., Zara F., Tumbarello F., Urru E., Melis A., Casula L. (2021). Multidimensional Impact of Mediterranean Diet on IBD Patients. Inflamm. Bowel Dis..

[78] Marsh A., Chachay V., Banks M., Okano S., Hartel G., Radford-Smith G. (2024). A Pilot Randomized Controlled Trial Investigating the Effects of an Anti-Inflammatory Dietary Pattern on Disease Activity, Symptoms and Microbiota Profile in Adults with Inflammatory Bowel Disease. Eur. J. Clin. Nutr..

[79] Lewis J.D., Sandler R.S., Brotherton C., Brensinger C., Li H., Kappelman M.D., Daniel S.G., Bittinger K., Albenberg L., Valentine J.F. (2021). A Randomized Trial Comparing the Specific Carbohydrate Diet to a Mediterranean Diet in Adults with Crohn’s Disease. Gastroenterology.

[80] Merra G., Noce A., Marrone G., Cintoni M., Tarsitano M.G., Capacci A., De Lorenzo A. (2020). Influence of Mediterranean Diet on Human Gut Microbiota. Nutrients.

[81] Martínez-González M.A., Gea A., Ruiz-Canela M. (2019). The Mediterranean Diet and Cardiovascular Health. Circ. Res..

[82] Schicho R., Marsche G., Storr M. (2015). Cardiovascular Complications in Inflammatory Bowel Disease. Curr. Drug Targets.

[83] Hashash J.G., Elkins J., Lewis J.D., Binion D.G. (2024). AGA Clinical Practice Update on Diet and Nutritional Therapies in Patients With Inflammatory Bowel Disease: Expert Review. Gastroenterology.

[84] Haas S.V., Haas M.P. (1955). The Treatment of Celiac Disease with the Specific Carbohydrate Diet; Report on 191 Additional Cases. Am. J. Gastroenterol..

[85] Dixon L.J., Kabi A., Nickerson K.P., McDonald C. (2015). Combinatorial Effects of Diet and Genetics on Inflammatory Bowel Disease Pathogenesis. Inflamm. Bowel Dis..

[86] Suskind D.L., Wahbeh G., Gregory N., Vendettuoli H., Christie D. (2014). Nutritional Therapy in Pediatric Crohn Disease: The Specific Carbohydrate Diet. J. Pediatr. Gastroenterol. Nutr..

[87] Obih C., Wahbeh G., Lee D., Braly K., Giefer M., Shaffer M.L., Nielson H., Suskind D.L. (2016). Specific Carbohydrate Diet for Pediatric Inflammatory Bowel Disease in Clinical Practice Within an Academic IBD Center. Nutrition.

[88] Kakodkar S., Farooqui A.J., Mikolaitis S.L., Mutlu E.A. (2015). The Specific Carbohydrate Diet for Inflammatory Bowel Disease: A Case Series. J. Acad. Nutr. Diet..

[89] Suskind D.L., Cohen S.A., Brittnacher M.J., Wahbeh G., Lee D., Shaffer M.L., Braly K., Hayden H.S., Klein J., Gold B. (2018). Clinical and Fecal Microbial Changes with Diet Therapy in Active Inflammatory Bowel Disease. J. Clin. Gastroenterol..

[90] Kaplan H.C., Opipari-Arrigan L., Yang J., Schmid C.H., Schuler C.L., Saeed S.A., Braly K.L., Chang F., Murphy L., Dodds C.M. (2022). Personalized Research on Diet in Ulcerative Colitis and Crohn’s Disease: A Series of N-of-1 Diet Trials. Am. J. Gastroenterol..

[91] Suskind D.L., Wahbeh G., Cohen S.A., Damman C.J., Klein J., Braly K., Shaffer M., Lee D. (2016). Patients Perceive Clinical Benefit with the Specific Carbohydrate Diet for Inflammatory Bowel Disease. Dig. Dis. Sci..

[92] Lorenz-Meyer H., Bauer P., Nicolay C., Schulz B., Purrmann J., Fleig W.E., Scheurlen C., Koop I., Pudel V., Carr L. (1996). Omega-3 Fatty Acids and Low Carbohydrate Diet for Maintenance of Remission in Crohn’s Disease: A Randomized Controlled Multicenter Trial. Scand. J. Gastroenterol..

[93] Ritchie J.K., Wadsworth J., Lennard-Jones J.E., Rogers E. (1987). Controlled Multicentre Therapeutic Trial of an Unrefined Carbohydrate, Fibre Rich Diet in Crohn’s Disease. Br. Med. J. Clin. Res. Ed..

[94] Kakodkar S., Mikolaitis S., Engen P., Mutlu E. (2013). The Bacterial Microbiome of IBD Patients on the Specific Carbohydrate Diet (SCD): 1828. Off. J. Am. Coll. Gastroenterol. ACG.

[95] Gibson P.R., Yao C.K., Halmos E.P. (2024). Review Article: Evidence-Based Dietary Management of Inflammatory Bowel Disease. Aliment. Pharmacol. Ther..

[96] Braly K., Williamson N., Shaffer M.L., Lee D., Wahbeh G., Klein J., Giefer M., Suskind D.L. (2017). Nutritional Adequacy of the Specific Carbohydrate Diet in Pediatric Inflammatory Bowel Disease. J. Pediatr. Gastroenterol. Nutr..

[97] Wellens J., Sabino J., Vanuytsel T., Tack J., Vermeire S. (2025). Recent Advances in Clinical Practice: Mastering the Challenge-Managing IBS Symptoms in IBD. Gut.

[98] Oka P., Parr H., Barberio B., Black C.J., Savarino E.V., Ford A.C. (2020). Global Prevalence of Irritable Bowel Syndrome According to Rome III or IV Criteria: A Systematic Review and Meta-Analysis. Lancet Gastroenterol. Hepatol..

[99] Fairbrass K.M., Costantino S.J., Gracie D.J., Ford A.C. (2020). Prevalence of Irritable Bowel Syndrome-Type Symptoms in Patients with Inflammatory Bowel Disease in Remission: A Systematic Review and Meta-Analysis. Lancet Gastroenterol. Hepatol..

[100] Bertin L., Zanconato M., Crepaldi M., Marasco G., Cremon C., Barbara G., Barberio B., Zingone F., Savarino E.V. (2024). The Role of the FODMAP Diet in IBS. Nutrients.

[101] Singh P., Chey S.W., Nee J., Eswaran S., Lembo A., Chey W.D., Dietary Therapy in IBS Working Group (2025). Is a Simplified, Less Restrictive Low FODMAP Diet Possible? Results from a Double-Blind, Pilot Randomized Controlled Trial. Clin. Gastroenterol. Hepatol. Off. Clin. Pract. J. Am. Gastroenterol. Assoc..

[102] Bodini G., Zanella C., Crespi M., Lo Pumo S., Demarzo M.G., Savarino E., Savarino V., Giannini E.G. (2019). A Randomized, 6-Wk Trial of a Low FODMAP Diet in Patients with Inflammatory Bowel Disease. Nutrition.

[103] Pedersen N., Ankersen D.V., Felding M., Wachmann H., Végh Z., Molzen L., Burisch J., Andersen J.R., Munkholm P. (2017). Low-FODMAP Diet Reduces Irritable Bowel Symptoms in Patients with Inflammatory Bowel Disease. World J. Gastroenterol..

[104] Peng Z., Yi J., Liu X. (2022). A Low-FODMAP Diet Provides Benefits for Functional Gastrointestinal Symptoms but Not for Improving Stool Consistency and Mucosal Inflammation in IBD: A Systematic Review and Meta-Analysis. Nutrients.

[105] Cox S.R., Lindsay J.O., Fromentin S., Stagg A.J., McCarthy N.E., Galleron N., Ibraim S.B., Roume H., Levenez F., Pons N. (2020). Effects of Low FODMAP Diet on Symptoms, Fecal Microbiome, and Markers of Inflammation in Patients with Quiescent Inflammatory Bowel Disease in a Randomized Trial. Gastroenterology.

[106] Uno Y. (2020). Why a Low FODMAP Diet Was Ineffective for IBS Symptoms in Quiescent Crohn’s Disease. Gastroenterology.

[107] Halmos E.P. (2016). A Low FODMAP Diet in Patients with Crohn’s Disease. J. Gastroenterol. Hepatol..

[108] Halmos E.P., Christophersen C.T., Bird A.R., Shepherd S.J., Muir J.G., Gibson P.R. (2016). Consistent Prebiotic Effect on Gut Microbiota With Altered FODMAP Intake in Patients with Crohn’s Disease: A Randomised, Controlled Cross-Over Trial of Well-Defined Diets. Clin. Transl. Gastroenterol..

[109] Zhan Y., Zhan Y.-A., Dai S.-X. (2018). Is a Low FODMAP Diet Beneficial for Patients with Inflammatory Bowel Disease? A Meta-Analysis and Systematic Review. Clin. Nutr. Edinb. Scotl..

[110] Elhusseiny M.H., Amine A.K., Salem O.E., Tayel D.I., Elsayed E.A. (2018). Low FODMAP Diet in Egyptian Patients with Crohn’s Disease in Remission Phase with Functional Gastrointestinal Symptoms. JGH Open Open Access J. Gastroenterol. Hepatol..

[111] Moreau L.A., Ford A.C., Brookes M.J., Graca S., Guthrie E., Hartley S., Houghton L., Kemp K., Kennedy N.A., McKenzie Y. (2025). Management of Diarrhoea in Patients with Stable Ulcerative Colitis with Low FODMAP Diet, Amitriptyline, Ondansetron or Loperamide: The MODULATE RCT. Health Technol. Assess. Winch. Engl..

[112] Staudacher H.M., Whelan K. (2017). The Low FODMAP Diet: Recent Advances in Understanding Its Mechanisms and Efficacy in IBS. Gut.

[113] Pessarelli T., Sorge A., Elli L., Costantino A. (2022). The Low-FODMAP Diet and the Gluten-Free Diet in the Management of Functional Abdominal Bloating and Distension. Front. Nutr..

[114] Vandeputte D., Joossens M. (2020). Effects of Low and High FODMAP Diets on Human Gastrointestinal Microbiota Composition in Adults with Intestinal Diseases: A Systematic Review. Microorganisms.

[115] So D., Loughman A., Staudacher H.M. (2022). Effects of a Low FODMAP Diet on the Colonic Microbiome in Irritable Bowel Syndrome: A Systematic Review with Meta-Analysis. Am. J. Clin. Nutr..

[116] Gwioździk W., Krupa-Kotara K., Całyniuk B., Helisz P., Grajek M., Głogowska-Ligus J. (2022). Traditional, Vegetarian, or Low FODMAP Diets and Their Relation to Symptoms of Eating Disorders: A Cross-Sectional Study Among Young Women in Poland. Nutrients.

[117] Staudacher H.M., Ralph F.S.E., Irving P.M., Whelan K., Lomer M.C.E. (2020). Nutrient Intake, Diet Quality, and Diet Diversity in Irritable Bowel Syndrome and the Impact of the Low FODMAP Diet. J. Acad. Nutr. Diet..

[118] Singh P., Tuck C., Gibson P.R., Chey W.D. (2022). The Role of Food in the Treatment of Bowel Disorders: Focus on Irritable Bowel Syndrome and Functional Constipation. Am. J. Gastroenterol..

[119] Black C.J., Staudacher H.M., Ford A.C. (2022). Efficacy of a Low FODMAP Diet in Irritable Bowel Syndrome: Systematic Review and Network Meta-Analysis. Gut.

[120] Olendzki B.C., Silverstein T.D., Persuitte G.M., Ma Y., Baldwin K.R., Cave D. (2014). An Anti-Inflammatory Diet as Treatment for Inflammatory Bowel Disease: A Case Series Report. Nutr. J..

[121] Keshteli A.H., Valcheva R., Nickurak C., Park H., Mandal R., van Diepen K., Kroeker K.I., van Zanten S.V., Halloran B., Wishart D.S. (2022). Anti-Inflammatory Diet Prevents Subclinical Colonic Inflammation and Alters Metabolomic Profile of Ulcerative Colitis Patients in Clinical Remission. Nutrients.

[122] Shafiee N.H., Manaf Z.A., Mokhtar N.M., Raja Ali R.A. (2021). Anti-Inflammatory Diet and Inflammatory Bowel Disease: What Clinicians and Patients Should Know?. Intest. Res..

[123] Fackelmann G., Manghi P., Carlino N., Heidrich V., Piccinno G., Ricci L., Piperni E., Arrè A., Bakker E., Creedon A.C. (2025). Gut Microbiome Signatures of Vegan, Vegetarian and Omnivore Diets and Associated Health Outcomes across 21,561 Individuals. Nat. Microbiol..

[124] Sidhu S.R.K., Kok C.W., Kunasegaran T., Ramadas A. (2023). Effect of Plant-Based Diets on Gut Microbiota: A Systematic Review of Interventional Studies. Nutrients.

[125] Ross F.C., Patangia D., Grimaud G., Lavelle A., Dempsey E.M., Ross R.P., Stanton C. (2024). The Interplay Between Diet and the Gut Microbiome: Implications for Health and Disease. Nat. Rev. Microbiol..

[126] Liu G.X.H., Day A.S. (2024). Plant-Based Diets for Inflammatory Bowel Disease: What Is the Evidence?. Inflamm. Bowel Dis..

[127] Chiba M., Abe T., Tsuda H., Sugawara T., Tsuda S., Tozawa H., Fujiwara K., Imai H. (2010). Lifestyle-Related Disease in Crohn’s Disease: Relapse Prevention by a Semi-Vegetarian Diet. World J. Gastroenterol. WJG.

[128] Chen J., Sun Y., Dan L., Wellens J., Yuan S., Yang H., Tong T.Y.N., Cross A.J., Papadimitriou N., Meyer A. (2025). Composition of Plant-Based Diets and the Incidence and Prognosis of Inflammatory Bowel Disease: A Multinational Retrospective Cohort Study. Lancet Reg. Health—Eur..

[129] Ahrens A.P., Culpepper T., Saldivar B., Anton S., Stoll S., Handberg E.M., Xu K., Pepine C., Triplett E.W., Aggarwal M. (2021). A Six-Day, Lifestyle-Based Immersion Program Mitigates Cardiovascular Risk Factors and Induces Shifts in Gut Microbiota, Specifically Lachnospiraceae, Ruminococcaceae, Faecalibacterium Prausnitzii: A Pilot Study. Nutrients.

[130] Malhotra A., Lakade A. (2025). Analytical Review on Nutritional Deficiencies in Vegan Diets: Risks, Prevention, and Optimal Strategies. J. Am. Nutr. Assoc..

[131] Teigen L.M., Geng Z., Sadowsky M.J., Vaughn B.P., Hamilton M.J., Khoruts A. (2019). Dietary Factors in Sulfur Metabolism and Pathogenesis of Ulcerative Colitis. Nutrients.

[132] Stummer N., Feichtinger R.G., Weghuber D., Kofler B., Schneider A.M. (2023). Role of Hydrogen Sulfide in Inflammatory Bowel Disease. Antioxidants.

[133] Rowan F.E., Docherty N.G., Coffey J.C., O’Connell P.R. (2009). Sulphate-Reducing Bacteria and Hydrogen Sulphide in the Aetiology of Ulcerative Colitis. Br. J. Surg..

[134] Day A.S., Yao C.K., Costello S.P., Ruszkiewicz A., Andrews J.M., Gibson P.R., Bryant R.V. (2022). Therapeutic Potential of the 4 Strategies to SUlfide-REduction (4-SURE) Diet in Adults with Mild to Moderately Active Ulcerative Colitis: An Open-Label Feasibility Study. J. Nutr..

[135] Sarbagili-Shabat C., Albenberg L., Van Limbergen J., Pressman N., Otley A., Yaakov M., Wine E., Weiner D., Levine A. (2021). A Novel UC Exclusion Diet and Antibiotics for Treatment of Mild to Moderate Pediatric Ulcerative Colitis: A Prospective Open-Label Pilot Study. Nutrients.

[136] Leibovitzh H., Sarbagili Shabat C., Hirsch A., Zittan E., Mentella M.C., Petito V., Cohen N.A., Ron Y., Fliss Isakov N., Pfeffer J. (2024). Faecal Transplantation for Ulcerative Colitis from Diet Conditioned Donors Followed by Dietary Intervention Results in Favourable Gut Microbial Profile Compared to Faecal Transplantation Alone. J. Crohns Colitis.

[137] Khalil N.A., Walton G.E., Gibson G.R., Tuohy K.M., Andrews S.C. (2014). In Vitro Batch Cultures of Gut Microbiota from Healthy and Ulcerative Colitis (UC) Subjects Suggest That Sulphate-Reducing Bacteria Levels Are Raised in UC and by a Protein-Rich Diet. Int. J. Food Sci. Nutr..

[138] Sandall A.M., Cox S.R., Lindsay J.O., Gewirtz A.T., Chassaing B., Rossi M., Whelan K. (2020). Emulsifiers Impact Colonic Length in Mice and Emulsifier Restriction Is Feasible in People with Crohn’s Disease. Nutrients.

[139] Fitzpatrick J.A., Gibson P.R., Taylor K.M., Anderson E.J., Friedman A.B., Ardalan Z.S., Smith R.L., Halmos E.P. (2025). Clinical Trial: The Effects of Emulsifiers in the Food Supply on Disease Activity in Crohn’s Disease: An Exploratory Double-Blinded Randomised Feeding Trial. Aliment. Pharmacol. Ther..

[140] Bancil A., Rossi M., Sandall A., Cox S., Dalrymple K., Kelaiditis C., Buckley A., Burke S., Xu Y., Smith L. (2025). DOP097 Emulsifier Restriction Is an Effective Therapy for Active Crohn’s Disease: The ADDapt Trial—A Multi-Centre, Randomised, Double-Blind, Placebo-Controlled, Re-Supplementation Trial in 154 Patients. J. Crohns Colitis.

[141] Aharoni Frutkoff Y., Plotkin L., Shavit Z., Focht G., Livovsky J., Lev-Zion R., Ledder O., Assa A., Yogev D., Orlanski-Meyer E. (2025). OP02 Tasty & Healthy Flexible Diet Induces Clinical and Biological Remission in Children and Young Adults with Mild-Moderate Crohn’s Disease Similar to EEN: Results from the “TASTI-MM” Randomized, Physician-Blinded, Controlled Trial. J. Crohns Colitis.

[142] O’Neill B., Raggi P. (2020). The Ketogenic Diet: Pros and Cons. Atherosclerosis.

[143] Sampaio L.P.d.B. (2016). Ketogenic Diet for Epilepsy Treatment. Arq. Neuropsiquiatr..

[144] Dyńka D., Kowalcze K., Paziewska A. (2022). The Role of Ketogenic Diet in the Treatment of Neurological Diseases. Nutrients.

[145] Li S., Zhuge A., Wang K., Lv L., Bian X., Yang L., Xia J., Jiang X., Wu W., Wang S. (2021). Ketogenic Diet Aggravates Colitis, Impairs Intestinal Barrier and Alters Gut Microbiota and Metabolism in DSS-Induced Mice. Food Funct..

[146] Kong C., Yan X., Liu Y., Huang L., Zhu Y., He J., Gao R., Kalady M.F., Goel A., Qin H. (2021). Ketogenic Diet Alleviates Colitis by Reduction of Colonic Group 3 Innate Lymphoid Cells through Altering Gut Microbiome. Signal Transduct. Target. Ther..

[147] Norwitz N.G., Soto-Mota A. (2024). Case Report: Carnivore-Ketogenic Diet for the Treatment of Inflammatory Bowel Disease: A Case Series of 10 Patients. Front. Nutr..

[148] Rew L., Harris M.D., Goldie J. (2022). The Ketogenic Diet: Its Impact on Human Gut Microbiota and Potential Consequent Health Outcomes: A Systematic Literature Review. Gastroenterol. Hepatol. Bed Bench.

[149] Popiolek-Kalisz J. (2024). Ketogenic Diet and Cardiovascular Risk—State of the Art Review. Curr. Probl. Cardiol..

[150] Zingone F., Zanini A., Corazza G.R., Troncone R., Lenti M.V., Silano M. (2024). Chapter 12—Gluten Free Diet, Assessment of Its Adherence, and Quality of Life. Pediatric and Adult Celiac Disease.

[151] Marsilio I., Canova C., D’Odorico A., Ghisa M., Zingone L., Lorenzon G., Savarino E.V., Zingone F. (2020). Quality-of-Life Evaluation in Coeliac Patients on a Gluten-Free Diet. Nutrients.

[152] Schiepatti A., Maimaris S., Randazzo S., Maniero D., Biti R., Caio G., Lungaro L., Carroccio A., Seidita A., Scalvini D. (2024). Resilience in Adult Coeliac Patients on a Gluten-Free Diet: A Cross-Sectional Multicentre Italian Study. Nutrients.

[153] Zingone F., Bartalini C., Siniscalchi M., Ruotolo M., Bucci C., Morra I., Iovino P., Ciacci C. (2017). Alterations in Diets of Patients with Nonceliac Gluten Sensitivity Compared With Healthy Individuals. Clin. Gastroenterol. Hepatol. Off. Clin. Pract. J. Am. Gastroenterol. Assoc..

[154] Catassi C., Alaedini A., Bojarski C., Bonaz B., Bouma G., Carroccio A., Castillejo G., De Magistris L., Dieterich W., Di Liberto D. (2017). The Overlapping Area of Non-Celiac Gluten Sensitivity (NCGS) and Wheat-Sensitive Irritable Bowel Syndrome (IBS): An Update. Nutrients.

[155] Herfarth H.H., Martin C.F., Sandler R.S., Kappelman M.D., Long M.D. (2014). Prevalence of a Gluten-Free Diet and Improvement of Clinical Symptoms in Patients with Inflammatory Bowel Diseases. Inflamm. Bowel Dis..

[156] Zingone F., Bertin L., Maniero D., Palo M., Lorenzon G., Barberio B., Ciacci C., Savarino E.V. (2023). Myths and Facts about Food Intolerance: A Narrative Review. Nutrients.

[157] Haskey N., Gibson D.L. (2017). An Examination of Diet for the Maintenance of Remission in Inflammatory Bowel Disease. Nutrients.

[158] Charlebois A., Rosenfeld G., Bressler B. (2016). The Impact of Dietary Interventions on the Symptoms of Inflammatory Bowel Disease: A Systematic Review. Crit. Rev. Food Sci. Nutr..

[159] Levenstein S., Prantera C., Luzi C., D’Ubaldi A. (1985). Low Residue or Normal Diet in Crohn’s Disease: A Prospective Controlled Study in Italian Patients. Gut.

[160] Woolner J., Parker T., Kirby G., Hunter J. (1998). The Development and Evaluation of a Diet for Maintaining Remission in Crohn’s Disease. J. Hum. Nutr. Diet..

[161] Vanhauwaert E., Matthys C., Verdonck L., De Preter V. (2015). Low-Residue and Low-Fiber Diets in Gastrointestinal Disease Management. Adv. Nutr..

[162] Wedlake L., Slack N., Andreyev H.J.N., Whelan K. (2014). Fiber in the Treatment and Maintenance of Inflammatory Bowel Disease: A Systematic Review of Randomized Controlled Trials. Inflamm. Bowel Dis..

[163] Makki K., Deehan E.C., Walter J., Bäckhed F. (2018). The Impact of Dietary Fiber on Gut Microbiota in Host Health and Disease. Cell Host Microbe.

[164] Sonnenburg E.D., Sonnenburg J.L. (2014). Starving Our Microbial Self: The Deleterious Consequences of a Diet Deficient in Microbiota-Accessible Carbohydrates. Cell Metab..

[165] Ferenc K., Jarmakiewicz-Czaja S., Filip R. (2022). Components of the Fiber Diet in the Prevention and Treatment of IBD-An Update. Nutrients.

[166] Hallert C., Björck I., Nyman M., Pousette A., Grännö C., Svensson H. (2003). Increasing Fecal Butyrate in Ulcerative Colitis Patients by Diet: Controlled Pilot Study. Inflamm. Bowel Dis..

[167] James S.L., Christophersen C.T., Bird A.R., Conlon M.A., Rosella O., Gibson P.R., Muir J.G. (2015). Abnormal Fibre Usage in UC in Remission. Gut.

[168] Fritsch J., Garces L., Quintero M.A., Pignac-Kobinger J., Santander A.M., Fernández I., Ban Y.J., Kwon D., Phillips M.C., Knight K. (2021). Low-Fat, High-Fiber Diet Reduces Markers of Inflammation and Dysbiosis and Improves Quality of Life in Patients with Ulcerative Colitis. Clin. Gastroenterol. Hepatol..

[169] Brotherton C.S., Taylor A.G., Bourguignon C., Anderson J.G. (2014). A High-Fiber Diet May Improve Bowel Function and Health-Related Quality of Life in Patients with Crohn Disease. Gastroenterol. Nurs. Off. J. Soc. Gastroenterol. Nurses Assoc..

[170] Brotherton C.S., Martin C.A., Long M.D., Kappelman M.D., Sandler R.S. (2016). Avoidance of Fiber Is Associated with Greater Risk of Crohn’s Disease Flare in a 6-Month Period. Clin. Gastroenterol. Hepatol. Off. Clin. Pract. J. Am. Gastroenterol. Assoc..

[171] Serrano Fernandez V., Seldas Palomino M., Laredo-Aguilera J.A., Pozuelo-Carrascosa D.P., Carmona-Torres J.M. (2023). High-Fiber Diet and Crohn’s Disease: Systematic Review and Meta-Analysis. Nutrients.

[172] Narula N., Wong E.C.L., Moayyedi P., Reinisch W., Marshall J.K. (2022). Pilot Study of an Elimination Diet in Adults with Mild to Moderate Crohn’s Disease. Eur. J. Gastroenterol. Hepatol..

[173] Bentz S., Hausmann M., Piberger H., Kellermeier S., Paul S., Held L., Falk W., Obermeier F., Fried M., Schölmerich J. (2010). Clinical Relevance of IgG Antibodies against Food Antigens in Crohn’s Disease: A Double-Blind Cross-Over Diet Intervention Study. Digestion.

[174] Rajendran N., Kumar D. (2011). Food-Specific IgG4-Guided Exclusion Diets Improve Symptoms in Crohn’s Disease: A Pilot Study. Colorectal Dis..

[175] Aydinlar E.I., Dikmen P.Y., Tiftikci A., Saruc M., Aksu M., Gunsoy H.G., Tozun N. (2013). IgG-Based Elimination Diet in Migraine plus Irritable Bowel Syndrome. Headache.

[176] Zar S., Mincher L., Benson M.J., Kumar D. (2005). Food-Specific IgG4 Antibody-Guided Exclusion Diet Improves Symptoms and Rectal Compliance in Irritable Bowel Syndrome. Scand. J. Gastroenterol..

[177] Atkinson W., Sheldon T.A., Shaath N., Whorwell P.J. (2004). Food Elimination Based on IgG Antibodies in Irritable Bowel Syndrome: A Randomised Controlled Trial. Gut.

[178] Ostrowska L., Wasiluk D., Lieners C.F.J., Gałęcka M., Bartnicka A., Tveiten D. (2021). Igg Food Antibody Guided Elimination-Rotation Diet Was More Effective than FODMAP Diet and Control Diet in the Treatment of Women with Mixed IBS—Results from an Open Label Study. J. Clin. Med..

[179] Singh P., Chey W.D., Takakura W., Cash B.D., Lacy B.E., Quigley E.M.M., Randall C.W., Lembo A. (2025). A Novel, IBS-Specific IgG ELISA-Based Elimination Diet in Irritable Bowel Syndrome: A Randomized, Sham-Controlled Trial. Gastroenterology.

[180] Svolos V., Hansen R., Nichols B., Quince C., Ijaz U.Z., Papadopoulou R.T., Edwards C.A., Watson D., Alghamdi A., Brejnrod A. (2019). Treatment of Active Crohn’s Disease With an Ordinary Food-Based Diet That Replicates Exclusive Enteral Nutrition. Gastroenterology.

[181] Wright R., Truelove S.C. (1965). A Controlled Therapeutic Trial of Various Diets in Ulcerative Colitis. Br. Med. J..

[182] Strisciuglio C., Giannetti E., Martinelli M., Sciorio E., Staiano A., Miele E. (2013). Does Cow’s Milk Protein Elimination Diet Have a Role on Induction and Maintenance of Remission in Children with Ulcerative Colitis?. Acta Paediatr..

[183] Albenberg L., Brensinger C.M., Wu Q., Gilroy E., Kappelman M.D., Sandler R.S., Lewis J.D. (2019). A Diet Low in Red and Processed Meat Does Not Reduce Rate of Crohn’s Disease Flares. Gastroenterology.

[184] Lomer M.C., Harvey R.S., Evans S.M., Thompson R.P., Powell J.J. (2001). Efficacy and Tolerability of a Low Microparticle Diet in a Double Blind, Randomized, Pilot Study in Crohn’s Disease. Eur. J. Gastroenterol. Hepatol..

[185] Lomer M.C.E., Grainger S.L., Ede R., Catterall A.P., Greenfield S.M., Cowan R.E., Vicary F.R., Jenkins A.P., Fidler H., Harvey R.S. (2005). Lack of Efficacy of a Reduced Microparticle Diet in a Multi-Centred Trial of Patients with Active Crohn’s Disease. Eur. J. Gastroenterol. Hepatol..

[186] Bhattacharyya S., Shumard T., Xie H., Dodda A., Varady K.A., Feferman L., Halline A.G., Goldstein J.L., Hanauer S.B., Tobacman J.K. (2017). A Randomized Trial of the Effects of the No-Carrageenan Diet on Ulcerative Colitis Disease Activity. Nutr. Healthy Aging.

[187] Sun V., Grant M., Wendel C.S., McMullen C.K., Bulkley J.E., Altschuler A., Ramirez M., Baldwin C.M., Herrinton L.J., Hornbrook M.C. (2015). Dietary and Behavioral Adjustments to Manage Bowel Dysfunction After Surgery in Long-Term Colorectal Cancer Survviors. Ann. Surg. Oncol..

[188] Croagh C., Shepherd S.J., Berryman M., Muir J.G., Gibson P.R. (2007). Pilot Study on the Effect of Reducing Dietary FODMAP Intake on Bowel Function in Patients without a Colon. Inflamm. Bowel Dis..

[189] Ardalan Z.S., Yao C.K., Green K., Probert C., Gill P.A., Rosella S., Muir J.G., Sparrow M.P., Gibson P.R. (2023). A Novel Monash Pouch Diet in Patients with an Ileoanal Pouch Is Tolerable and Has Favorable Metabolic Luminal Effects. JGH Open Open Access J. Gastroenterol. Hepatol..

[190] Shen B. (2024). Pouchitis: Pathophysiology and Management. Nat. Rev. Gastroenterol. Hepatol..

[191] Bertin L., Nasrallah M., Redavid C., Bonazzi E., Maniero D., Lorenzon G., De Barba C., Facchin S., Scarpa M., Ruffolo C. (2024). Risk Factors and Postoperative Outcomes in Pouchitis Following Restorative Proctocolectomy: An 18-Year Single-Center Study. Gastroenterol. Insights.

[192] Ianco O., Tulchinsky H., Lusthaus M., Ofer A., Santo E., Vaisman N., Dotan I. (2013). Diet of Patients after Pouch Surgery May Affect Pouch Inflammation. World J. Gastroenterol. WJG.

[193] Godny L., Maharshak N., Reshef L., Goren I., Yahav L., Fliss-Isakov N., Gophna U., Tulchinsky H., Dotan I. (2019). Fruit Consumption Is Associated with Alterations in Microbial Composition and Lower Rates of Pouchitis. J. Crohns Colitis.

[194] Godny L., Reshef L., Pfeffer-Gik T., Goren I., Yanai H., Tulchinsky H., Gophna U., Dotan I. (2020). Adherence to the Mediterranean Diet Is Associated with Decreased Fecal Calprotectin in Patients with Ulcerative Colitis after Pouch Surgery. Eur. J. Nutr..

[195] Ardalan Z.S., Livingstone K.M., Polzella L., Avakian J., Rohani F., Sparrow M.P., Gibson P.R., Yao C.K. (2023). Perceived Dietary Intolerances, Habitual Intake and Diet Quality of Patients with an Ileoanal Pouch: Associations with Pouch Phenotype (and Behaviour). Clin. Nutr. Edinb. Scotl..

[196] McLaughlin S.D., Culkin A., Cole J., Clark S.K., Tekkis P.P., Ciclitira P.J., Nicholls R.J., Whelan K. (2013). Exclusive Elemental Diet Impacts on the Gastrointestinal Microbiota and Improves Symptoms in Patients with Chronic Pouchitis. J. Crohns Colitis.

[197] Fliss Isakov N., Kornblum J., Zemel M., Cohen N.A., Hirsch A., Maharshak N. (2023). The Effect of the Crohn’s Disease Exclusion Diet on Patients with Pouch Inflammation: An Interventional Pilot Study. Clin. Gastroenterol. Hepatol. Off. Clin. Pract. J. Am. Gastroenterol. Assoc..

[198] Adamina M., Minozzi S., Warusavitarne J., Buskens C., Chaparro M., Verstockt B., Kopylov U., Agrawal M., Allocca M., Atreya R. (2024). ECCO Guidelines on Therapeutics in Crohn’s Disease: Surgical Treatment. J. Crohns Colitis.

[199] Rocha R., de J Santos G., Santana G. (2021). Influence of Nutritional Status in the Postoperative Period of Patients with Inflammatory Bowel Disease. World J. Gastrointest. Pharmacol. Ther..

[200] Adamina M., Gerasimidis K., Sigall-Boneh R., Zmora O., de Buck van Overstraeten A., Campmans-Kuijpers M., Ellul P., Katsanos K., Kotze P.G., Noor N. (2020). Perioperative Dietary Therapy in Inflammatory Bowel Disease. J. Crohns Colitis.

[201] Kehlet H., Wilmore D.W. (2002). Multimodal Strategies to Improve Surgical Outcome. Am. J. Surg..

[202] Tidadini F., Bonne A., Trilling B., Quesada J.-L., Sage P.-Y., Foote A., Arvieux C., Faucheron J.-L. (2022). Effect of Implementation of Enhanced Recovery after Surgery (ERAS) Protocol and Risk Factors on 3-Year Survival after Colorectal Surgery for Cancer-a Retrospective Cohort of 1001 Patients. Int. J. Color. Dis..

[203] Ni X., Jia D., Chen Y., Wang L., Suo J. (2019). Is the Enhanced Recovery After Surgery (ERAS) Program Effective and Safe in Laparoscopic Colorectal Cancer Surgery? A Meta-Analysis of Randomized Controlled Trials. J. Gastrointest. Surg. Off. J. Soc. Surg. Aliment. Tract.

[204] Fiorindi C., Cuffaro F., Piemonte G., Cricchio M., Addasi R., Dragoni G., Scaringi S., Nannoni A., Ficari F., Giudici F. (2020). Effect of Long-Lasting Nutritional Prehabilitation on Postoperative Outcome in Elective Surgery for IBD. Clin. Nutr..

[205] Godala M., Gaszyńska E., Walczak K., Małecka-Wojciesko E. (2024). An Evaluation of the Usefulness of Selected Screening Methods in Assessing the Risk of Malnutrition in Patients with Inflammatory Bowel Disease. Nutrients.

[206] Balestrieri P., Ribolsi M., Guarino M.P.L., Emerenziani S., Altomare A., Cicala M. (2020). Nutritional Aspects in Inflammatory Bowel Diseases. Nutrients.

[207] Shariff S., Moran G., Grimes C., Cooney R.M. (2021). Current Use of EEN in Pre-Operative Optimisation in Crohn’s Disease. Nutrients.

[208] Brennan G.T., Ha I., Hogan C., Nguyen E., Jamal M.M., Bechtold M.L., Nguyen D.L. (2018). Does Preoperative Enteral or Parenteral Nutrition Reduce Postoperative Complications in Crohn’s Disease Patients: A Meta-Analysis. Eur. J. Gastroenterol. Hepatol..

[209] Costa-Santos M.P., Palmela C., Torres J., Ferreira A., Velho S., Ourô S., Glória L., Gordo I., Maio R., Cravo M. (2020). Preoperative Enteral Nutrition in Adults with Complicated Crohn’s Disease: Effect on Disease Outcomes and Gut Microbiota. Nutrition.

[210] Gatti S., Galeazzi T., Franceschini E., Annibali R., Albano V., Verma A.K., De Angelis M., Lionetti M.E., Catassi C. (2017). Effects of the Exclusive Enteral Nutrition on the Microbiota Profile of Patients with Crohn’s Disease: A Systematic Review. Nutrients.

[211] Collins S., Castelan V.C., Wernick R., Banty A., Gwarnicki C., Solomon T., Martirosyan L., Roberts P., Wise A., Hampton M. (2024). Effects of nutrition status and perioperative nutrition supplement completion on postoperative outcomes in patients with inflammatory bowel disease undergoing surgery. Inflamm. Bowel Dis..

[212] Beattie A.H., Prach A.T., Baxter J.P., Pennington C.R. (2000). A Randomised Controlled Trial Evaluating the Use of Enteral Nutritional Supplements Postoperatively in Malnourished Surgical Patients. Gut.

[213] Vanderstappen J., Hoekx S., Bislenghi G., D’Hoore A., Verstockt B., Sabino J. (2024). Preoperative Optimization: Review on Nutritional Assessment and Strategies in IBD. Curr. Opin. Pharmacol..

[214] Gianotti L., Sandini M., Romagnoli S., Carli F., Ljungqvist O. (2020). Enhanced Recovery Programs in Gastrointestinal Surgery: Actions to Promote Optimal Perioperative Nutritional and Metabolic Care. Clin. Nutr. Edinb. Scotl..

[215] Heaton K.W., Thornton J.R., Emmett P.M. (1979). Treatment of Crohn’s Disease with an Unrefined-Carbohydrate, Fibre-Rich Diet. Br. Med. J..

[216] Loy L., Petronio L., Marcozzi G., Bezzio C., Armuzzi A. (2024). Dietary Fiber in Inflammatory Bowel Disease: Are We Ready to Change the Paradigm?. Nutrients.

[217] Cavalcanti E., Marra A., Mileti A., Donghia R., Curlo M., Mastronardi M. (2024). Nutritional Management in Stricturing Crohn’s Disease: A Pilot Study. Nutrients.

[218] Hu D., Ren J., Wang G., Li G., Liu S., Yan D., Gu G., Zhou B., Wu X., Chen J. (2014). Exclusive Enteral Nutritional Therapy Can Relieve Inflammatory Bowel Stricture in Crohn’s Disease. J. Clin. Gastroenterol..

[219] Marafini I., Salvatori S., Troncone E., Scarozza P., Fantini E., Monteleone G. (2020). No Effect of a Liquid Diet in the Management of Patients with Stricturing Crohn’s Disease. Int. J. Color. Dis..

[220] Fitzpatrick J.A., Melton S.L., Yao C.K., Gibson P.R., Halmos E.P. (2022). Dietary Management of Adults with IBD—The Emerging Role of Dietary Therapy. Nat. Rev. Gastroenterol. Hepatol..

[221] Critch J., Day A.S., Otley A., King-Moore C., Teitelbaum J.E., Shashidhar H., NASPGHAN IBD Committee (2012). Use of Enteral Nutrition for the Control of Intestinal Inflammation in Pediatric Crohn Disease. J. Pediatr. Gastroenterol. Nutr..

[222] Urlep D., Benedik E., Brecelj J., Orel R. (2020). Partial Enteral Nutrition Induces Clinical and Endoscopic Remission in Active Pediatric Crohn’s Disease: Results of a Prospective Cohort Study. Eur. J. Pediatr..

[223] Szczubełek M., Pomorska K., Korólczyk-Kowalczyk M., Lewandowski K., Kaniewska M., Rydzewska G. (2021). Effectiveness of Crohn’s Disease Exclusion Diet for Induction of Remission in Crohn’s Disease Adult Patients. Nutrients.

[224] Haskey N., Estaki M., Ye J., Shim R.K., Singh S., Dieleman L.A., Jacobson K., Gibson D.L. (2023). A Mediterranean Diet Pattern Improves Intestinal Inflammation Concomitant with Reshaping of the Bacteriome in Ulcerative Colitis: A Randomised Controlled Trial. J. Crohns Colitis.

[225] Amrousy D.E., Elashry H., Salamah A., Maher S., Abd-Elsalam S.M., Hasan S. (2022). Adherence to the Mediterranean Diet Improved Clinical Scores and Inflammatory Markers in Children with Active Inflammatory Bowel Disease: A Randomized Trial. J. Inflamm. Res..

[226] Suskind D.L., Lee D., Kim Y.-M., Wahbeh G., Singh N., Braly K., Nuding M., Nicora C.D., Purvine S.O., Lipton M.S. (2020). The Specific Carbohydrate Diet and Diet Modification as Induction Therapy for Pediatric Crohn’s Disease: A Randomized Diet Controlled Trial. Nutrients.

[227] Cohen S.A., Gold B.D., Oliva S., Lewis J., Stallworth A., Koch B., Eshee L., Mason D. (2014). Clinical and Mucosal Improvement With Specific Carbohydrate Diet in Pediatric Crohn Disease. J. Pediatr. Gastroenterol. Nutr..

[228] Roediger W.E.W. (1998). Decreased Sulphur Aminoacid Intake in Ulcerative Colitis. Lancet.

[229] Prince A.C., Myers C.E., Joyce T., Irving P., Lomer M., Whelan K. (2016). Fermentable Carbohydrate Restriction (Low FODMAP Diet) in Clinical Practice Improves Functional Gastrointestinal Symptoms in Patients with Inflammatory Bowel Disease. Inflamm. Bowel Dis..

[230] Massironi S., Viganò C., Palermo A., Pirola L., Mulinacci G., Allocca M., Peyrin-Biroulet L., Danese S. (2023). Inflammation and Malnutrition in Inflammatory Bowel Disease. Lancet Gastroenterol. Hepatol..

[231] Altomare R., Damiano G., Abruzzo A., Palumbo V.D., Tomasello G., Buscemi S., Lo Monte A.I. (2015). Enteral Nutrition Support to Treat Malnutrition in Inflammatory Bowel Disease. Nutrients.

[232] Nakahigashi M., Yamamoto T., Sacco R., Hanai H., Kobayashi F. (2016). Enteral Nutrition for Maintaining Remission in Patients with Quiescent Crohn’s Disease: Current Status and Future Perspectives. Int. J. Color. Dis..

[233] Yamamoto T., Shiraki M., Nakahigashi M., Umegae S., Matsumoto K. (2013). Enteral Nutrition to Suppress Postoperative Crohn’s Disease Recurrence: A Five-Year Prospective Cohort Study. Int. J. Color. Dis..

[234] Christensen C., Knudsen A., Arnesen E.K., Hatlebakk J.G., Sletten I.S., Fadnes L.T. (2024). Diet, Food, and Nutritional Exposures and Inflammatory Bowel Disease or Progression of Disease: An Umbrella Review. Adv. Nutr..

[235] Cawood A.L., Elia M., Stratton R.J. (2012). Systematic Review and Meta-Analysis of the Effects of High Protein Oral Nutritional Supplements. Ageing Res. Rev..

[236] Madini N., Vincenti A., Beretta A., Santero S., Viroli G., Cena H. (2024). Addressing Inflammaging and Disease-Related Malnutrition: Adequacy of Oral Nutritional Supplements in Clinical Care. Nutrients.

[237] Narula N., Dhillon A., Zhang D., Sherlock M.E., Tondeur M., Zachos M. (2018). Enteral Nutritional Therapy for Induction of Remission in Crohn’s Disease. Cochrane Database Syst. Rev..

[238] Zachos M., Tondeur M., Griffiths A.M. (2007). Enteral Nutritional Therapy for Induction of Remission in Crohn’s Disease. Cochrane Database Syst. Rev..

[239] Akobeng A.K., Thomas A.G. (2007). Enteral Nutrition for Maintenance of Remission in Crohn’s Disease. Cochrane Database Syst. Rev..

[240] Akobeng A.K., Zhang D., Gordon M., MacDonald J.K. (2018). Enteral Nutrition for Maintenance of Remission in Crohn’s Disease. Cochrane Database Syst. Rev..

[241] Cohen L.J., Cho J.H., Gevers D., Chu H. (2019). Genetic Factors and the Intestinal Microbiome Guide Development of Microbe-Based Therapies for Inflammatory Bowel Diseases. Gastroenterology.

[242] Gevers D., Kugathasan S., Denson L.A., Vázquez-Baeza Y., Van Treuren W., Ren B., Schwager E., Knights D., Song S.J., Yassour M. (2014). The Treatment-Naive Microbiome in New-Onset Crohn’s Disease. Cell Host Microbe.

[243] Ananthakrishnan A.N., Whelan K., Allegretti J.R., Sokol H. (2025). Diet and Microbiome-Directed Therapy 2.0 for IBD. Clin. Gastroenterol. Hepatol. Off. Clin. Pract. J. Am. Gastroenterol. Assoc..

[244] Zhao Y., Chen Z., Dong R., Liu Y., Zhang Y., Guo Y., Yu M., Li X., Wang J. (2024). Multiomics Analysis Reveals the Potential Mechanism of High-Fat Diet in Dextran Sulfate Sodium-Induced Colitis Mice Model. Food Sci. Nutr..

[245] Olendzki B., Bucci V., Cawley C., Maserati R., McManus M., Olednzki E., Madziar C., Chiang D., Ward D.V., Pellish R. (2022). Dietary Manipulation of the Gut Microbiome in Inflammatory Bowel Disease Patients: Pilot Study. Gut Microbes.

[246] Vich Vila A., Imhann F., Collij V., Jankipersadsing S.A., Gurry T., Mujagic Z., Kurilshikov A., Bonder M.J., Jiang X., Tigchelaar E.F. (2018). Gut Microbiota Composition and Functional Changes in Inflammatory Bowel Disease and Irritable Bowel Syndrome. Sci. Transl. Med..

[247] Jacobs J.P., Lagishetty V., Hauer M.C., Labus J.S., Dong T.S., Toma R., Vuyisich M., Naliboff B.D., Lackner J.M., Gupta A. (2023). Multi-Omics Profiles of the Intestinal Microbiome in Irritable Bowel Syndrome and Its Bowel Habit Subtypes. Microbiome.

[248] Alghamdi A., Gerasimidis K., Blackburn G., Akinci D., Edwards C., Russell R.K., Watson D.G. (2018). Untargeted Metabolomics of Extracts from Faecal Samples Demonstrates Distinct Differences Between Paediatric Crohn’s Disease Patients and Healthy Controls but No Significant Changes Resulting from Exclusive Enteral Nutrition Treatment. Metabolites.

[249] Marques J.G., Schwerd T., Bufler P., Koletzko S., Koletzko B. (2022). Metabolic Changes during Exclusive Enteral Nutrition in Pediatric Crohn’s Disease Patients. Metabolomics Off. J. Metabolomic Soc..

[250] da Silva Barros V.J., Severo J.S., Mendes P.H.M., da Silva A.C.A., de Oliveira K.B.V., Parente J.M.L., Lima M.M., Neto E.M.M., Aguiar Dos Santos A., Tolentino Bento da Silva M. (2021). Effect of Dietary Interventions on Inflammatory Biomarkers of Inflammatory Bowel Diseases: A Systematic Review of Clinical Trials. Nutrition.

[251] Papastratis I., Konstantinidis D., Daras P., Dimitropoulos K. (2024). AI Nutrition Recommendation Using a Deep Generative Model and ChatGPT. Sci. Rep..

[252] Day A.S., Ballard T.M., Yao C.K., Gibson P.R., Bryant R.V. (2024). Food-Based Interventions as Therapy for Inflammatory Bowel Disease: Important Steps in Diet Trial Design and Reporting of Outcomes. Inflamm. Bowel Dis..

[253] Whitton C., Ramos-García C., Kirkpatrick S.I., Healy J.D., Dhaliwal S.S., Boushey C.J., Collins C.E., Rollo M.E., Kerr D.A. (2022). A Systematic Review Examining Contributors to Misestimation of Food and Beverage Intake Based on Short-Term Self-Report Dietary Assessment Instruments Administered to Adults. Adv. Nutr..

[254] Macdiarmid J., Blundell J. (1998). Assessing Dietary Intake: Who, What and Why of under-Reporting. Nutr. Res. Rev..

[255] Whelan K., Alexander M., Gaiani C., Lunken G., Holmes A., Staudacher H.M., Theis S., Marco M.L. (2024). Design and Reporting of Prebiotic and Probiotic Clinical Trials in the Context of Diet and the Gut Microbiome. Nat. Microbiol..

[256] Gormley I.C., Bai Y., Brennan L. (2020). Combining Biomarker and Self-Reported Dietary Intake Data: A Review of the State of the Art and an Exposition of Concepts. Stat. Methods Med. Res..

[257] Lee B.Y., Ordovás J.M., Parks E.J., Anderson C.A.M., Barabási A.-L., Clinton S.K., de la Haye K., Duffy V.B., Franks P.W., Ginexi E.M. (2022). Research Gaps and Opportunities in Precision Nutrition: An NIH Workshop Report. Am. J. Clin. Nutr..

[258] Crichton G.E., Howe P.R., Buckley J.D., Coates A.M., Murphy K.J., Bryan J. (2012). Long-Term Dietary Intervention Trials: Critical Issues and Challenges. Trials.

